# Unraveling epigenomic signatures and effectiveness of electroconvulsive therapy in treatment-resistant depression patients: a prospective longitudinal study

**DOI:** 10.1186/s13148-024-01704-z

**Published:** 2024-07-17

**Authors:** Rosana Carvalho Silva, Paolo Martini, Christa Hohoff, Stefania Mattevi, Marco Bortolomasi, Maria Abate, Valentina Menesello, Massimo Gennarelli, Bernhard T. Baune, Alessandra Minelli

**Affiliations:** 1 Department of Molecular and Translational Medicine, Biology and Genetic Division, University of Brescia, Viale Europa, 11, 25123 Brescia, Italy; 2 Department of Psychiatry and Psychotherapy, University of Münster, Münster, Germany; 3 Psychiatric Hospital “Villa Santa Chiara”, Verona, Italy; 4 Genetics Unit, IRCCS Istituto Centro San Giovanni di Dio Fatebenefratelli, Brescia, Italy; 5 Department of Psychiatry, Melbourne Medical School, University of Melbourne, Melbourne, Australia; 6 The Florey Institute of Neuroscience and Mental Health, The University of Melbourne, Parkville, VIC Australia

**Keywords:** Treatment-resistant depression, TRD, Methylome, Epigenome-wide association study, Epigenetic mechanisms, Electroconvulsive therapy, ECT, ECT mechanisms, Inflammation, Immune responses

## Abstract

**Background:**

Electroconvulsive therapy (ECT) benefits patients with treatment-resistant depression (TRD), but the underlying biological processes are unclear. We conducted an epigenome-wide association study in 32 TRD patients undergoing ECT to depict ECT-associated methylation changes. Illness severity and ECT outcomes were assessed with the Montgomery–Åsberg Depression Rating Scale at baseline (T0) and 1 month after its end (T1). Methylation was profiled at T0 and T1 with the Illumina Infinium Methylation EPIC BeadChip array.

**Results:**

Longitudinal T0–T1 analyses showed 3 differentially methylated probes (DMPs) with nominal *p* values ≤ 10^−5^, with 2 annotated in the genes *CYB5B* and *PVRL4*. Including covariates, we found 4 DMPs for symptoms variation, annotated in *FAM20C*, *EPB41*, *OTUB1* and *ADARB1*, and 3 DMPs for response status, with 2 annotated in *IQCE* and *FAM20C*. Regional analysis revealed 54 differentially methylated regions (DMRs) with nominal *p* value area ≤ 0.05, with 9 presenting adjusted *p*-value area ≤ 0.10, annotated in *MCF2L*, *SLC25A24*, *RUNX3*, *MIR637*, *FOXK2*, *FAM180B*, *POU6F1*, *ALS2CL* and *CCRL2*. Considering covariates, we found 21 DMRs for symptoms variation and 26 DMRs for response (nominal *p* value area ≤ 0.05), with 4 presenting adjusted *p*-value area ≤ 0.10 for response, annotated in *SNORD34*, *NLRP6*, *GALNT2* and *SFT2D3*. None remained significant after false discovery rate correction. Notably, *ADARB1* variants are associated with suicide attempt in patients with psychiatric disorders, and *SLC25A24* relates to conduct disorder. Several DMPs and DMRs are annotated in genes associated with inflammatory/immune processes. Longitudinal analyses on females (n = 22) revealed statistically significant DMRs (adjusted *p* value area ≤ 0.05) and trend-significant DMRs (adjusted *p* value area ≤ 0.07) for symptoms variation and response status, annotated in genes related to psychiatric disorders (*ZFP57*, *POLD4, TRIM10, GAS7, ADORA2A, TOLLIP*), trauma exposure (*RIPOR2*) and inflammatory/immune responses (*LAT*, *DLX4*, *POLD4*, *FAM30A, H19*). Pathway analysis on females revealed enrichment for transcriptional activity, growth factors, DNA maintenance, and immune pathways including *IRF7* and *IRF2*.

**Conclusion:**

Although no significant results were found for the whole cohort, the study provides insights into ECT-associated methylation changes, highlighting DMPs and DMRs related to ECT outcomes. Analyses on females revealed significant DMRs and pathways related to psychiatric disorders and inflammatory/immune processes.

**Supplementary Information:**

The online version contains supplementary material available at 10.1186/s13148-024-01704-z.

## Background

Electroconvulsive therapy (ECT) is a well-established treatment for patients with severe and treatment-resistant depression (TRD) [1, 2]. This therapy reaches response rates varying from 60 to 80%, remission rates of 50–60%, quicker clinical response when compared to pharmacological treatments, and reduced hospital length of stay and number of hospitalizations [2, 3].

The biological processes underlying ECT mechanisms of action are still largely unclear, and the study of molecular changes associated with therapeutic response to ECT in TRD patients enables a wider insight into the pathophysiology of severe depression and treatment response [4]. The majority of the studies present in the literature focused on effects on proteins, the few genetic studies conducted to date used a candidate gene method, and there is currently no genomic investigation of ECT response. Currently, expression studies that look at RNA or miRNA levels as well as epigenetic research are rare.

Concerning epigenetic mechanisms, such as DNA methylation, current research on ECT has usually focused on candidate gene approaches [5–7], but, given the diversity and complexity of the mechanisms involved in major depressive disorder (MDD) pathophysiology, a whole methylome approach has the potential to provide a wider picture of the disease neurobiology and treatment responses.

Few recent studies have been performed to investigate methylomic changes in depressed patients undergoing ECT [8, 9]. Analyzing DNA methylome in 12 TRD patients receiving ECT, Moschny and collaborators [8] found no significant differences in longitudinal global methylation measures or between responders and non-responders, but revealed eight genes that may be involved in ECT response, including *RNF213*, related to immunological mechanisms, angiogenesis and MDD. Using a similar approach in 34 depressed patients, Sirignano et al. [9] found significant differentially methylated cytosine-phosphate-guanine dinucleotide (CpG) sites associated with binary or continuous response to ECT, highlighting *TNKS*, associated with MDD and other psychiatric diseases, and *FKBP5*, which regulates the stress–response system being linked to psychiatric disorders, including MDD.

On these bases, we conducted an epigenome-wide association study (EWAS) in a cohort of TRD patients undergoing ECT sessions, aiming at identifying methylation changes associated with ECT outcomes, in order to find potential biomarkers related to antidepressant response and gain a deeper insight into ECT biological mechanisms of action.

## Methods

### Study participants and clinical assessment

Thirty-two TRD patients were voluntarily enrolled in the study. The diagnostic criterion for inclusion was a diagnosis of MDD according to the Diagnostic and Statistical Manual of Mental Disorders-IV (DSM-IV) classification system. The exclusion criteria were the following: (a) cognitive impairment or mental retardation; (b) history of bipolar disorder, schizophrenia or schizoaffective disorder; (c) primary diagnosis of substance abuse, alcohol abuse or dependency, obsessive compulsive disorder, personality disorder or PTSD; and (d) comorbidity with eating disorders; (e) comorbidity with alcohol and substance dependence; (f) neurological disorders (i.e., Parkinson's disease, multiple sclerosis, Alzheimer's and other dementias, epilepsy, stroke, brain tumors, traumatic conditions of the nervous system); (g) comorbidity with other severe medical illness and severe autoimmune diseases (i.e., cancers, Crohn's disease, rheumatoid arthritis (RA), scleroderma, psoriasis, myasthenia gravis, Sjögren syndrome, systemic lupus erythematosus); and (h) pregnancy. Patients were referred to the Psychiatric Hospital “Villa Santa Chiara” in Verona, Italy. The study was approved by the Ethics Committee for Clinical Trials of province of Verona and Rovigo (N: 4997/09.11.01). Participants received full explanation about study procedures and gave written informed consents to participate.

All the patients were evaluated as having TRD. TRD definition was the failure to respond to at least two trials with two or more classes of antidepressant drugs and to a trial with a tricyclic drug, corresponding to stage III or above, in accordance with the Thase and Rush staging system [10].

All the patients were scheduled to undergo ECT. ECT was performed three times a week, between 7:00 and 9:00 a.m., using a Thymatron DG (Somatics, Inc., Lake Bluff, IL, USA) with standard settings with a bipolar brief pulse square wave and bilateral electrode placement. The ECT procedure has been described in detail elsewhere [11]. The mean number of treatments received was 7.4 ± 2.2, and ECT treatment was completed on the basis of the clinical judgment of the treating physicians. Illness severity and the outcomes of ECT were assessed using the Montgomery–Åsberg Depression Rating Scale (MADRS) [12] before the treatment (T0) and about 1 month after the last ECT session (T1). Patients were considered as responders if the MADRS reduction was > 50% at T1. In addition, symptom improvement at both time points was defined as the % variation (Delta) of MADRS score compared to baseline computed as ((T1 score − T0 score) / T0 score) * 100.

### DNA extraction and methylation analysis

Fasting blood samples were collected at T0 and T1 using EDTA tube, and the DNA was extracted from whole blood samples using the Gentra Puregene Blood kit (Qiagen), according to the manufacturer’s instructions. DNA quantification and quality evaluation were performed through spectrophotometric analysis (NanoDrop 2000, Thermo Scientific). DNAs were pipetted on 96-well processing plates: same-subject T0 and T1 DNAs on the same plate; and between-subjects randomized based on sex and age on separate plates.

Methylation was profiled at T0 and T1 with Illumina Infinium Methylation EPIC BeadChip array (850 k) using HiScan array scanning systems (chips and scanner from Illumina, San Diego, CA).

Methylation levels were quantified after quality control and normalization using ChAMP R package. Probes with detection p-value cutoff below 0.01 and with a bead count less than 3 in at least 5% of patients were removed. We also removed probes containing single-nucleotide polymorphisms (SNPs) with minor allele frequency above 0.01 within 10 base pair (bp) of the single base extension position based on the list from Pidsley and collaborators [13]. Probes linked to X- and Y-chromosomes were removed. None of the samples has more than 10% of not available (NA), and all were retained for analysis. Normalization was performed on beta-values using Beta-Mixture Quantile (BMIQ) normalization as implemented in ChAMP R package. After normalization, beta-values were transformed to M-values to perform association [14].

### Statistical analysis

A mixed linear model approach implemented in the Limma R package was used to perform association analysis using patient as blocking factor. To adjust for confounding factors, we included white blood cell fractions estimated for granulocytes, monocytes, B cells, NK cells, CD4 + T cells and CD8 + T cells, and 2 principal components from control probes.

We estimated white blood cellular composition using the estimateCellCounts function from the R minfi package [15]. Principal component analysis (PCA) was performed on EPIC chip control probes to correct for technical artifacts. Optimal number of principal components to use was determined using the findPC R package [16].

Differentially methylated probes (DMPs) were reported at the suggestive threshold of *p* ≤ 10^−5^. Cytosine guanine dinucleotide (CpG) site annotation was performed using IlluminaHumanMethylationEPICanno.ilm10b2.hg19 R package (hg19 genome reference).

Differentially methylation region (DMR) analysis was performed using bumphunter R package. Single probe statistic was calculated using a univariate model with patient as blocking factor and the phenotype/feature of interest as explanatory variable. Probes were aggregated in clusters/regions with at least 8 probes, with maximum distance of 300 bp within each probe. Region *p* values were computed by permutation procedure over 250 permutations. Differentially methylated regions (DMRs) were considered those with adjusted *p* value ≤ 0.05, but also those with adjusted *p* value ≤ 0.1 are reported in the supplementary tables. DMRs were considered relevant with adjusted *p* value area ≤ 0.05.

Enrichment analysis was performed with enricher and enrichGO functions from clusterProfiler R package. Gene sets were obtained from graphite [17, 18], msigdbr and hsa-ord-db R packages. Significant enriched gene sets were considered as those with an adjusted *p* value ≤ 0.2. *P* values were adjusted using the Benjamini & Hochberg method unless otherwise stated.

## Results

The socio-demographical and clinical characteristics of the studied patients (n = 32) are depicted in Table 1. The mean age of the enrolled participants was 56.9 years (standard deviation of 14.3 years) and 68.7% were females. After ECT sessions, at the follow-up visit (T1), 23 patients were considered responders and 9 patients were classified as non-responders.

**Table 1﻿:** Patients’ demographical and clinical characteristics

	Whole cohort (n = 32)	Responders (n = 23)	Non-responders (n = 9)
Age in years, mean (SD)	56.9 (± 14.3)	56.9 (± 15.5)	57.0 (± 11.5)
Gender, n (% F)	22 (68.7)	18 (78.2)	4 (44.5)
Education in years, mean (SD)	8.0 (± 2.9)	8.0 (± 3.1)	8.6 (± 2.3)
BMI, mean (SD)	26.8 (± 5.3)	27.0 (± 5.8)	27.1 (± 4.0)
Smokers, n (%)	10 (31.2)	7 (30.4)	3 (33.4)
MADRS at T0, mean (SD)	33.4 (± 7.4)	33.0 (± 7.2)	34.1 (± 8.7)
MADRS at T1, mean (SD)	11.0 (± 10.0)	5.5 (± 3.9)	25.0 (± 6.1)
Delta% MADRS at T1, mean (SD)	65.9 (± 29.6)	82.2 (± 11.8)	24.2 (± 17.5)
Psychotic symptoms, n (%)	23 (71.8)	16 (69.5)	7 (77.8)
Recurrent MDD, n (%)	29 (90.6)	20 (86.9)	9 (100)
Comorbidity with personality disorders, n (%)	6 (18.8)	3 (33.3)	3 (13.0)
Comorbidity with anxiety disorders, n (%)	3 (9.4)	3 (13.0)	0 (0.0)
ECT sessions, mean (SD)	7.4 (2.2)	7.0 (1.7)	8.2 (3.2)

*Note: BMI:* body mass index, *ECT:* electroconvulsive therapy, *F:* females, *MADRS:* Montgomery–Åsberg depression rating scale, *MDD:* major depressive disorder, *SD:* standard deviation

### Single CpG site analysis

In order to evaluate possible epigenome-wide methylation changes before and after ECT sessions, we performed longitudinal analyses to evaluate DMPs between T0 and T1 in the whole cohort of patients. We identified 3 DMPs with nominal *p* values ≤ 10^−5^, with 2 annotated in the genes *CYB5B* and *PVRL4*. After FDR correction, none of these probes remained significant (Supplementary Table 1Sa). Additionally, we conducted T0–T1 analyses adding covariates into the model, i.e., clinical symptoms variations assessed by MADRS and response status. We found 4 DMPs when including the covariate clinical symptoms variations, annotated in the genes *FAM20C*, *EPB41*, *OTUB1* and *ADARB1* (Supplementary Table 1Sb), and 3 DMPs for the covariate response status at T1, with 2 annotated in the genes *IQCE* and *FAM20C* (Supplementary Table 1Sc), all with nominal *p* values ≤ 10^−5^. However, after FDR correction, none of these probes remained significant.

Separate longitudinal analyses were performed in the female cohort, consisting of 22 patients. We found 4 DMPs with nominal *p* values ≤ 10^−5^, with 3 annotated in the genes *CUL1*, *FOXK1* and *CYB5B*. After FDR correction, none of these probes remained significant (Supplementary Table 3Sa). Adding clinical symptoms variations to the model, we identified 7 DMPs with nominal *p* values ≤ 10^−5^, with 4 annotated in the genes *EIF3H*, *PHLPP2*, *FAM20C* and *DOCK4* (Supplementary Table 3Sb). Adding response status, we found 6 DMPs with nominal *p* values ≤ 10^−5^, with 5 annotated in the genes *MUC4*, *SEC16A*, *CDH4*, *MBD4* and *PARD3* (Supplementary Table 3Sc). After FDR correction, none of these probes remained significant.

### Differentially methylated regional analysis

To identify DMRs in longitudinal correlational analyses performed between T0 and T1, regional analyses were conducted in the entire cohort. The DMR analysis between T0 and T1 resulted in 54 DMRs with nominal *p* value area ≤ 0.05. From these regions, 9 presented adjusted *p* value area results ≤ 0.10, annotated in the genes *MCF2L*, *SLC25A24*, *RUNX3*, *MIR637*, *FOXK2*, *FAM180B*, *POU6F1*, *ALS2CL* and *CCRL2*, with none remaining significant after FDR adjustment (Supplementary Table 2Sa). Moreover, the first 6 regions presented trend-significant adjusted *p* value area results annotated in the following genes: *MCF2L*, *SLC25A24*, *RUNX3*, *MIR637*, *FOXK2* and *FAM180B*.

Separate longitudinal regional analyses were performed on the female cohort (n = 22). The DMR analysis between T0 and T1 identified 56 DMRs with adjusted *p* value ≤ 0.05. Among these regions, 7 had adjusted *p* value area results ≤ 0.10, annotated in the genes *MIR637*, *AVPI1*, *RUNX3*, *MCF2L*, *FOXK2*, *FAM124B* and *POU6F1*, but none remained significant after FDR adjustment (Supplementary Table 4Sa).

When considering the covariates into the model, we found several DMRs with nominal *p* value area ≤ 0.05: 21 DMRs when considering clinical symptoms variation as measured by MADRS (Supplementary Table 2Sb) and 26 DMRs for response status (Supplementary Table 2Sc). From these regions, 4 presented adjusted *p* value area results ≤ 0.10, when considering the covariate response status, annotated in the genes *SNORD34*, *NLRP6*, *GALNT2* and *SFT2D3*. However, after FDR correction none of these regions remained significant.

In the separate analyses in the female cohort, considering clinical symptom variation, we found 53 DMRs with nominal *p* value area ≤ 0.05. Among these regions, 5 had significant adjusted p-value area, annotated in the genes *ZFP57*, *LAT*, *DLX4*, *POT1* and *POLD4*. Additionally, 8 DMRs had adjusted *p* value area results ≤ 0.10, annotated in the genes *MCIDAS*, *OTX1*, *ARMC9*, *GDNF*, *NPRL2*, *FBXO27*, *ADORA2A* and *AQP1* (Supplementary Table 4Sb). When considering response status, we identified 49 DMRs with nominal *p* value area ≤ 0.05. Of these, 9 had significant adjusted *p* value area, annotated in the genes *KBTBD11*, *PPP1R14A*, *ARFGAP1*, *RIPOR2*, *FAM30A*, *DLX4*, *LAT*, *SNORD34* and *POLD4*. Additionally, 19 had adjusted *p* value area ≤ 0.10, annotated in the genes *H19*, *GALNT2*, *TRIM10*, *SFT2D3*, *MACROH2A1*, *GAS7*, *ADORA2A*, *INPP5D*, *TOLLIP*, *SLC35A4*, *SEPTIN9*, *POU2AF1*, *ZNF205-AS1*, *BHLHE40*, *C16orf54*, *POT1*, *TENM3*, *UBXN11* and *GNAS*, with the first 12 showing adjusted *p* value area results ≤ 0.07 (Supplementary Table 4Sc).

Table 2 shows the top 10 genes for the DMRs analyses in T0-T1, as well as analyses with the covariates just described above.

**Table 2:** Top 10 genes for differentially methylated regions (DMRs) in correlational analyses performed in T0-T1 for the whole cohort and for the female cohort. The Table shows a) the DMRs at T0-T1 and the DMRs analysed with covariates including b) clinical symptoms variations (assessed by delta MADRS) and c) response (reduction greater than 50% in MADRS score at T1). MADRS: Montgomery-Åsberg Depression Rating Scale. Statistically significant genes are highlighted in bold

*(a) T0–T1—Whole cohort*					
DMR	Main genes	CHR	*p* Value area	Adjusted *p* value area	Gene associated or predicted function
1	*MCF2L*	chr13	0.0001	0.0524	Encodes a guanine nucleotide exchange factor that interacts with the guanosine-5'-triphosphate (GTP)-bound Rac1 and plays a role in the Rho/Rac signaling pathways^1^ Associates with osteoarthritis [19]
2	*SLC25A24*	chr1	0.0002	0.0524	Encodes a carrier protein that transports adenosine triphosphate (ATP)-Magnesium exchanging it for phosphate^1^ Associated with conduct disorder trait [20]
3	*RUNX3*	chr1	0.0002	0.0524	Encodes a member of the runt domain-containing family of transcription factors, functions as tumor suppressor gene^1^
4	*MIR637*	chr19	0.0002	0.0524	Micro-RNA gene associated with squamous cell carcinoma, renal cell carcinoma and systemic lupus erythematosus^1^
5	*FOXK2*	chr17	0.0002	0.0524	Involved in the regulation of viral, cellular promoter elements and various types of cancers [21]
6	*FAM180B*	chr11	0.0002	0.0524	Predicted to be associated with leprosy^1^
7	*POU6F1*	chr12	0.0005	0.0870	Associated with corticotropin-releasing hormone expression regulation, neuroplasticity [22] and lung adenocarcinoma [23]
8	*ALS2CL*	chr3	0.0005	0.0870	Enables identical protein binding activity, associated with deafness and megalencephalic leukoencephalopathy^1^
9	*CCRL2*	chr3	0.0006	0.0870	Encodes a chemokine receptor like protein, a seven transmembrane protein and related to chemokine receptor 1^1^ Involved in immune processes and lung cancer growth [24]
10	*TEX55*	chr3	0.0008	0.1036	Predicted to be active in nucleus, involved in cryptorchidism^1^

*(a) T0–T1—Female cohort*					
DMR	Main genes	CHR	*p* Value area	Adjusted *p* value area	Gene associated or predicted function
1	*MIR637*	chr19	0.0001	0.0781	Micro-RNA gene associated with squamous cell carcinoma, renal cell carcinoma and systemic lupus erythematosus^1^
2	*AVPI1*	chr10	0.0002	0.0781	Associated with melanoma cells [25] and alcohol dependence [26]
3	*RUNX3*	chr1	0.0003	0.0781	Encodes a member of the runt domain-containing family of transcription factors, functions as tumor suppressor gene^1^
4	*MCF2L*	chr13	0.0003	0.0781	Associated with different types of cancer [27, 28] and osteoarthritis^1^
5	*FOXK2*	chr17	0.0003	0.0781	Involved in the regulation of viral, cellular promoter elements and various types of cancers [21]
6	*FAM124B*	chr2	0.0004	0.0781	Associated with anorexia nervosa [29], leukemia and other types of cancer [30]
7	*POU6F1*	chr12	0.0004	0.0824	Associated with corticotropin-releasing hormone expression regulation, neuroplasticity [22] and lung adenocarcinoma [23]
8	*DAZL*	chr3	0.0013	0.1330	Associated with schizophrenia [31]
9	*IL3*	chr5	0.0014	0.1330	Related to inflammatory and immune processes^1^
10	*SPECC1*	chr17	0.0016	0.1330	Associated with some cancer types^1^

*(b) T0–T1 (Delta MADRS)—Whole cohort*					
DMR	Main genes	CHR	*p* Value area	Adjusted *p* value area	Gene function
1	*ARFGAP1*	chr20	0.0006	0.1283	Encodes a GTPase-activating protein, associated with the Golgi apparatus^1^
2	*DYNLRB1*	chr20	0.0004	0.1283	Encodes a cytoplasmic protein that binds intermediate chain proteins and interacts with transforming growth factor-beta^1^
3	*DLK1*	chr14	0.0001	0.1283	Encodes a transmembrane protein that contains multiple epidermal growth factor repeats functioning as a regulator of cell growth^1^
4	*SGF29*	chr16	0.0005	0.1283	Component of the SAGA (Spt-Ada-Gcn5 acetyltransferase) transcriptional coactivator complex, related to chromatin organization^1^
5	*PCDHGA5*	chr5	0.0005	0.1283	Member of the protocadherin gamma gene cluster, involved in immunoglobulin-like organization^1^
6	*CACFD1*	chr9	0.0013	0.1371	Predicted to be involved in vesicle-mediated transport^1^
7	*ECI1*	chr16	0.0008	0.1317	Encodes a member of the hydratase/isomerase superfamily, a key mitochondrial enzyme involved in beta-oxidation of unsaturated fatty acids^1^
8	*POT1*	chr7	0.0015	0.1371	Member of the telombin family and encodes a nuclear protein involved in telomere maintenance^1^
9	*LEPROTL1*	chr8	0.0010	0.1371	Predicted to be involved in late endosome to vacuole transport via multivesicular body sorting pathway and negative regulation of growth hormone receptor signaling pathway^1^
10	*ARMC9*	chr2	0.0013	0.1371	Predicted to be involved in cilium assembly and positive regulation of smoothened signaling pathway^1^

*(b) T0–T1 (Delta MADRS)—Female cohort*					
DMR	Main genes	CHR	*p* Value area	Adjusted *p* value area	Gene function
**1**	***ZFP57***	**chr6**	**0.0000**	**0.0313**	**Associated with autism spectrum **[32]** and PTSD symptoms **[33]
**2**	***LAT***	**chr16**	**0.0001**	**0.0313**	**Associated with immune processes**^**1**^
**3**	***DLX4***	**chr17**	**0.0001**	**0.0313**	**Associated with various cancer types**^**1**^
**4**	***POT1***	**chr7**	**0.0001**	**0.0313**	**Member of the telombin family and encodes a nuclear protein involved in telomere maintenance**^**1**^
**5**	***POLD4***	**chr11**	**0.0002**	**0.0448**	**Associated with schizophrenia **[34]** and various cancer types**
6	*MCIDAS*	chr5	0.0005	0.0844	Related to the respiratory epithelium and infectious diseases^1^
7	*OTX1*	chr2	0.0005	0.0844	Associated with autism spectrum disorders [35]
8	*ARMC9*	chr2	0.0006	0.0873	Predicted to be involved in cilium assembly and positive regulation of smoothened signaling pathway^1^
9	*GDNF*	chr5	0.0008	0.0985	Related to the neurotrophic system^1^
10	*NPRL2*	chr3	0.0009	0.0985	Associated with epilepsy^1^

*(c) T0–T1 (Response status)—Whole cohort*					
DMR	Main genes	CHR	*p* Value area	Adjusted *p* value area	Gene function
1	*SNORD34*	chr19	0.0002	0.0754	Predicted to act upstream of or within glucose metabolic process; insulin secretion; and reactive oxygen species metabolic process^1^
2	*NLRP6*	chr11	0.0003	0.0754	Involved in the arginine vasopressin-mediated regulation of renal salt–water balance^1^ Maintains tissue homeostasis, involved in inflammatory processes [36]
3	*GALNT2*	chr1	0.0003	0.0754	Encodes a member of the glycosyltransferase 2 protein family, associated in O-linked glycosylation of the immunoglobulin A1 hinge region^1^ Involved in several types of metabolic diseases and cancer pathology [37]
4	*SFT2D3*	chr2	0.0002	0.0754	Predicted to be involved in protein transport and vesicle-mediated transport^1^
5	*ARFGAP1*	chr20	0.0005	0.1067	Encodes a GTPase-activating protein, associated with the Golgi apparatus^1^
6	*FAM30A*	chr14	0.0013	0.1587	Affiliated with the long noncoding lncRNA class, associated with gallbladder cancer^1^
7	*EFNA1*	chr1	0.0024	0.1928	Comprises a subfamily of receptor protein tyrosine kinases, implicated in mediating developmental events in the nervous system and in erythropoiesis^1^
8	*STRADA*	chr17	0.0011	0.1587	Involved in catalytic activity, mutations result in polyhydramnios, megalencephaly and symptomatic epilepsy^1^
9	*GMCL1*	chr2	0.0012	0.1587	Encodes a nuclear envelope protein involved in spermatogenesis^1^
10	*PPP1R14A*	chr19	0.0013	0.1587	Encodes a protein that is inhibitor of smooth muscle myosin phosphatase^1^

*(c) T0–T1 (Response status)—Female cohort*					
DMR	Main genes	CHR	*p* Value area	Adjusted *p* value area	Gene function
**1**	***KBTBD11***	**chr8**	**0.0002**	**0.0416**	**Associated with deafness**^**1**^
**2**	***PPP1R14A***	**chr19**	**0.0002**	**0.0416**	**Encodes a protein that is inhibitor of smooth muscle myosin phosphatase**^**1**^
**3**	***ARFGAP1***	**chr20**	**0.0003**	**0.0416**	**Encodes a GTPase-activating protein, associated with the Golgi apparatus**^**1**^
**4**	***RIPOR2***	**chr6**	**0.0003**	**0.0416**	**Associated with altered methylation in trauma exposure **[38]** and some cancer types**^**1**^
**5**	***FAM30A***	**chr14**	**0.0003**	**0.0416**	**Affiliated with the long noncoding lncRNA class, associated with gallbladder cancer**^**1**^
**6**	***DLX4***	**chr17**	**0.0003**	**0.0416**	**Associated with some cancer types, neurogenesis and Alzheimer disease **[39]
**7**	***LAT***	**chr16**	**0.0003**	**0.0416**	**Related to inflammatory/immune processes**^**1**^
**8**	***SNORD34***	**chr19**	**0.0003**	**0.0416**	**Predicted to act upstream of or within glucose metabolic process; insulin secretion; and reactive oxygen species metabolic process**^**1**^
**9**	***POLD4***	**chr11**	**0.0003**	**0.0417**	**Associated with schizophrenia **[34]** and some cancer types**^**1**^
10	*H19*	chr11	0.0005	0.0561	Associated with cognitive impairment, Alzheimer disease [40] and some cancer types^1^

Note: ^1^Provided by Alliance of Genome Resources/The Human Gene Database (https://www.genecards.org/)

Table 3 provides a summary of the main DMPs, DMRs and gene sets known to be associated with inflammatory and immune responses, psychiatric disorders, neuroplasticity and other biological pathways.

**Table 3﻿:** Summary of genes in differentially methylated probes (DMPs), differentially methylated regions (DMRs) and pathway enrichment analyses in correlational analyses performed in T0–T1, as well as with covariates including clinical symptoms variations and response status. Statistically significant genes are highlighted in bold

*Single CpG site analysis—Whole cohort*		
	**Genes**	**Main known associations**
Genes in DMPs related to inflammatory and immune processes	*PVRL4*	Breast and ovarian cancer
	*EPB41*	Liver and lung cancer
	*OTUB1*	Pro-carcinogenic roles
	*FAM20C*	Bladder, brain and stomach cancer
Genes in DMPs related to psychiatric disorders	*ADARB1*	Bipolar disorder, MDD, schizophrenia

*Single CpG site analysis—Female cohort*		
	**Genes**	**Main known associations**
Genes in DMPs related to inflammatory and immune processes	*FOXK1*	Some types of cancer
	*CYB5B*	Some types of cancer
	*PHLPP2*	Some types of cancer
	*FAM20C*	Bladder, brain and stomach cancer
Genes in DMPs related to psychiatric disorders	*EIF3H*	Autism spectrum
	*DOCK4*	Schizophrenia and autism

*Regional analysis—Whole cohort*		
	**Genes**	**Main known associations**
Genes in DMRs related to inflammatory and immune processes	*MCF2L*	Inflammatory processes and osteoarthritis
	*RUNX3*	Oncogenic and tumor suppression functions
	*MIR637*	Renal cell carcinoma
	*FOXK2*	Various cancer cell types
	*POU6F1*	Lung cancer
	*CCRL2*	Lung cancer
	*NLRP6*	Inflammatory processes
	*GALNT2*	Various cancer cell types
Genes in DMRs related to psychiatric disorders	*SLC25A24*	Conduct disorder
Genes in DMRs related to neuroplasticity	*POU6F1*	Neurogenesis and neuroplasticity

*Regional analysis—Female cohort*		
	**Genes**	**Main known associations**
Genes in DMRs related to inflammatory and immune processes	***LAT***	Immune processes
	***FAM30A***	Gallbladder cancer
	***DLX4***	Various cancer cell types
	*RUNX3*	Oncogenic and tumor suppression functions
	*MCF2L*	Inflammatory processes and osteoarthritis
	*FOXK2*	Various cancer cell types
	*MIR637*	Renal cell carcinoma
	*AVPI1*	Melanoma
Genes in DMRs related to psychiatric disorders	***ZFP57***	Autism spectrum and PTSD symptoms
	***POLD4***	Schizophrenia
	***RIPOR2***	Altered in trauma exposure
	*TRIM10*	Schizophrenia
	*GAS7*	Schizophrenia
	*ADORA2A*	Anxiety disorders
	*AVPI1*	Alcohol dependence
	*DAZL*	Schizophrenia
	*FAM124B*	Anorexia nervosa
Genes in DMRs related to neuroplasticity	***DLX4***	Neurogenesis and Alzheimer disease
	*POU6F1*	Neurogenesis and neuroplasticity

*Gene set and biological pathway analysis—Female cohort*	
	**Main pathways**
Gene ontology biological processes	Oxidative stress
Gene ontology molecular functions	Regulation of transcriptional activity
	Growth factor activity
	Glutamate receptor binding and other binding functions
Gene ontology cellular components	Neuron projection membrane and axolemma terms
KEGG pathways	Alcoholism pathway
Reactome pathways	DNA maintenance
MSigDB	MYC targets V2
Transcription factor targets	Targets of *IRF7*, *IRF2*, *AUTS2*

### Gene and pathway enrichment analysis in the female cohort

Gene enrichment analyses in the female cohort were conducted on the 40, 56 and 63 genes located on the DMRs (adjusted *p* value ≤ 0.05) identified in the T0–T1 analyses performed without covariates, including clinical symptoms variations (assessed by delta MADRS) at T1, and including response status at T1, respectively. We found no relevant enrichments for the T0–T1 analysis without covariates. This may be due to the small number of genes overlapping DMRs in this comparison.

When analyzing DMR-associated genes obtained by including clinical symptoms variations, we observed enrichment for several pathways (Supplementary Fig. 1). We found genes associated with gene ontology (GO) molecular functions (MF) such as regulation of transcriptional activity and growth factor (Supplementary Fig. 1A; adjusted *p* value ≤ 0.2). Using KEGG pathways, we observed enrichment for alcoholism pathways (Supplementary Fig. 1B; *p* value = 0.005 and adjusted *p* value 0.14). When using Reactome pathways, we observed 83 significant pathways (Supplementary Fig. 1C; adjusted *p* value ≤ 0.2). Overall, most significant pathways can be referred to DNA maintenance. Notably, we observed enrichment for the hallmark “MYC Targets V2” (Supplementary Fig. 1D; adjusted *p* value 0.18). Examining the Transcription factor targets, we identified 7 gene sets and 12 genes with an adjusted *p* value ≤ 0.2 in the Legacy (Supplementary Fig. 2A) and GTRD (Supplementary Fig. 2B) gene sets as defined by the Molecular Signature Database. Enrichments include targets of *IRF7*, *IRF2* and *AUTS2*.

In the analysis including response status at T1, we found 58, 8 and 2 enriched terms with adjusted *p* values ≤ 0.2 for GO biological processes (BP), GO MF and GO cellular components (CC), respectively. Among the biological processes in enriched terms, we observed several processes related to oxidative stress (Supplementary Fig. 3A). Among molecular functions, we observed “glutamate receptor binding” (adjusted *p* value 0.13) and other DNA binding functions (Supplementary Fig. 3B). Regarding cellular components, we identified “neuron projection membrane” and “axolemma” terms, both with adjusted *p* values ≤ 0.05 (Supplementary Fig. 3C).

## Discussion

Our study investigated epigenome-wide longitudinal changes in a cohort of TRD patients undergoing ECT. The aim was to identify methylation changes associated with ECT treatment, with the goal of finding potential biomarkers related to treatment outcomes. While we identified some DMPs and DMRs related to ECT outcomes in the T0–T1 analyses, the results did not remain statistically significant after FDR correction.

In the single CpG site analysis, considering only probes annotated in genes, we found 2 DMPs in the *CYB5B* and *PVRL4* genes. When including covariates in the model, we identified 4 DMPs, annotated in the genes *FAM20C*, *EPB41*, *OTUB1* and *ADARB1*, when considering clinical symptom variations. Additionally, we found 2 DMPs, annotated in the genes *IQCE* and *FAM20C*, when including response status at T1. Variants in the *ADARB1* gene, located in a region probably linked to familial bipolar disorder and whose product has an action in the editing of the pre-mRNA of glutamate receptor B subunit, have been associated with suicide attempt vulnerability, along with recent stressful life events and childhood trauma in patients with MDD, bipolar disorder and schizophrenia [41]. Additionally, altered expression levels of ADARB1 have been observed in postmortem brain samples of major depressive suicide victims [42].

In the longitudinal regional analysis, we found 9 DMRs annotated in the genes *MCF2L*, *SLC25A24*, *RUNX3*, *MIR637*, *FOXK2*, *FAM180B*, *POU6F1*, *ALS2CL* and *CCRL2*. When considering covariates, we found 4 DMRs annotated in genes (*SNORD34*, *NLRP6*, *GALNT2* and *SFT2D3*) when adding response status. Notably, the gene *SLC25A24*, a member of a solute carrier gene family, is associated with adenosine triphosphate-mediated calcium buffering at the mitochondrial matrix, potentially involved in protecting cells against oxidative stress-induced cell death. In adolescent girls with conduct disorder, elevated levels of callous–unemotional traits correlated with decreased SLC25A24 gene expression, while in typically developing girls, conduct disorder traits were positively associated with SLC25A24 gene expression [20]].

To date, the literature on this theme is scarce, with few studies conducted to investigate methylomic changes in depressed patients undergoing ECT [8, 9, 43]. Moschny et al. [8] examined epigenome-wide DNA methylation changes associated with ECT treatment response in 12 TRD patients. They found no global DNA methylation differences between measured time points (before and after the first and last ECT session) or between ECT responders (8 patients) and non-responders (4 patients). No significant effects were observed for time, response or the interaction between time and response in the global DNA methylation analysis considering ECT response. In single CpG site analyses, they identified 5 protein-coding candidate genes implicated in ECT response (*RNF175*, *RNF213*, *TBC1D14*, *TMC5* and *WSCD1*). Additionally, they observed differences between ECT responder groups within 3 gene regions encoding for long noncoding RNA transcripts (*AC018685.2*, *AC098617.1* and *CLCN3P1*) and reported changes in DNA methylation of 2 CpG sites, located within *AQP10* and *TRERF1*, during the treatment course. Sirignano et al. [9] studied the effects of ECT on epigenome-wide DNA methylation levels associated with response in 34 patients with TRD. They measured global DNA methylation levels before the first and after the last ECT session, identifying one differentially methylated CpG site annotated in *TNKS* associated with ECT binary response and one differentially methylated CpG site annotated in *FKBP5* associated with continuous response. Regional analyses identified two DMRs on chromosomes 8 and 20 associated with continuous response. A recent study combined neuroimaging and transcriptomic gene expression analyses in MDD patients receiving ECT found that increased gray matter volume correlated with higher expression levels of MDD risk genes including *CNR1*, *HTR1A*, *MAOA*, *PDE1A* and *SST*, as well as ECT related genes of *BDNF*, *DRD2*, *APOE*, *P2RX7* and *TBC1D14* [43].

Although we did not find similar DMPs and/or DMRs, our results indicate possible mechanisms of action and response-related biomarkers in ECT treatment that should be replicated in subsequent studies. The comparison of our results with the available literature is limited by some factors. Moschny et al. [8] utilized the TruSeq Methyl Capture EPIC Library Kit for methylation analysis, while we used the Illumina Infinium Methylation EPIC BeadChip, similar to Sirignano et al. [9]. The use of diverse microarrays covering different CpG sites and regions certainly limits the comparison of results. Additionally, Moschny and Sirignano and their colleagues [8, 9], employed a slightly different ECT protocol compared to ours. They applied right unilateral electrical stimulation or bilateral electrode placement in case of non-response, while we exclusively applied bilateral electrode placement in all ECT sessions. Also, the studies differ in the measured time points. Moschny et al. [8] collected blood samples at four time points, immediately before and 15 min after the first and last ECT sessions, with treatment administered for up to four weeks. Sirignano et al. [9] measured DNA methylation at baseline and 1–7 days after the last ECT session. In our study, we measured DNA methylation at two time points: at baseline and one month after the last ECT session. Evaluating antidepressant effects of ECT treatment over a 4-week period following the last session may allow for the detection of midterm epigenome-wide changes, extending beyond immediate- or short-term effects. Previous studies investigating longitudinal epigenome-wide effects of treatments for MDD or PTSD assessed methylation changes over more extended periods [44, 45]. In our study, we aimed to capture lasting changes induced by ECT by conducting a longer follow-up period, in contrast to previous EWAS on ECT that focused on more immediate effects [8, 9]. Lastly, Sirignano et al. [9] used Hamilton Depression Rating Scale for evaluating symptoms changes and treatment response, while in our study and in Moschny et al. [8] one MADRS score was used. These differences should be considered when comparing the results.

In our findings, we have identified numerous DMPs and DMRs annotated in genes associated with inflammatory and immune processes. For instance, among the DMPs annotated in genes, the *PVRL4* gene is linked to breast tumor cell lines, lung and ovarian cancer [46]. *FAM20C* is widely expressed across various cancers, including bladder urothelial carcinoma, brain lower grade glioma and stomach adenocarcinoma [47]. Dysregulation of *EPB41* is implicated in hepatocellular carcinoma [48] and lung cancer [49]. Finally, *OTUB1* has a pro-carcinogenic role and is known for its association with immune responses [50]. Among the DMRs annotated in genes, *MCF2L*, which regulates neurotrophin-3 induced cell migration, is associated with inflammatory processes and osteoarthritis [19]. *RUNX3* plays a role in immunity and has been implicated in both oncogenic and tumor suppressive functions [51]. *MIR637* is down-regulated in most cancers and up-regulated in clear cell renal cell carcinoma [52]. *FOXK2* plays a crucial role in the transcriptional regulation of various cancer types [21]. *POU6F1* is associated with corticotropin-releasing hormone expression regulation, neuroplasticity [22] and the proliferation of lung adenocarcinoma [23]. *CCRL2* is involved in immune processes and lung cancer growth [24]. The *NLRP6* inflammasome is critical in maintaining tissue homeostasis, while improper inflammasome activation may contribute to the development of multiple diseases [36]. *GALNT2* is involved in several types of metabolic diseases and cancer pathology [37]. In summary, our findings suggest alterations in mechanisms such as neurogenesis and neuroinflammatory immune response, which have been proposed to be related to the mechanisms of action of ECT [4, 53, 54].

Given the higher prevalence of MDD in females, who typically present with greater disease severity and different responses to antidepressant treatment compared to males [55], we conducted a separate analysis considering only the female cohort. In regional analyses between the two time points, we found no significant DMRs, although some genes known to be associated with inflammatory and immune processes (*MIR637*, *RUNX3*, *AVPI1*, *MCF2L*, *FOXK2*, *POU6F1*) were noted. Moreover, *FAM124B* is related to anorexia nervosa [29], and *AVPI1* is associated with alcohol dependence [26]. In the regional analyses including clinical symptoms variation as a covariate, we identified several significant DMRs annotated in notable genes. For example, *ZFP57* is associated with autism spectrum disorder [32] and PTSD symptoms [33], while *POLD4* is linked to schizophrenia [34]. Other significant genes, such as *LAT*, *DLX4* and *POLD4*, are related to inflammatory/immune processes and various cancer types. The longitudinal analyses revealed the highest number of significant DMRs when we included the covariate response status, uncovering several interesting genes. For instance, altered methylation of *RIPOR2* was associated with trauma exposure [38], and *POLD4* is linked to schizophrenia risk [34]. Other significant genes, including *RIPOR2*, *FAM30A*, *DLX4* and *LAT*, are associated with inflammatory/immune processes and various cancer etiologies. Additionally, *DLX4* is linked to neurogenesis and Alzheimer's disease [39]. In the same covariate analyses, when considering trend-significant associations, we identified genes such as *TRIM10* and *GAS7*, associated with schizophrenia [56, 57], *MACROH2A1*, related to autism-like behaviors [58], and *ADORA2A*, associated with anxiety disorders [59]. Many trend-significant genes, including *H19*, *GALNT2*, *TRIM10*, *MACROH2A1*, *GAS7*, *ADORA2A*, *INPP5D*, *SEPTIN9* and *POU2AF1*, are associated with inflammatory processes and various cancer types. *SLC35A4* is related to epileptic encephalopathy [60], while *H19*, *ADORA2A* and *INPP5D* are linked to cognitive impairment and Alzheimer's disease [40, 61, 62]. Most interestingly, although the *TOLLIP* gene showed a trend-significant association, it is related to inflammation in patients with MDD and stress-related disorders [63] and is more highly expressed in patients with MDD compared to controls, regardless of childhood trauma exposure [64].

Contrasting the female group with the whole patient group, we found some DMRs with gene overlaps, albeit with different levels of significance. For example, in the T0–T1 regional analyses, the genes *MCF2L*, *RUNX3*, *MIR637* and *FOXK2* were trend-significant for the whole group but nonsignificant in females. Considering clinical symptoms variation, *POT1* was nonsignificant in the whole group but significant in women, whereas *ARMC9* was nonsignificant in both groups. Regarding response status, the DMRs annotated in the genes *SNORD34*, *ARFGAP1*, *FAM30A* and *PPP1R14A* were all nonsignificant in the whole group but significant in women. These divergences may highlight important sex-based differences in TRD pathophysiology and ECT treatment response, underscoring the importance of conducting research considering sex-related differences.

Gene and pathway analysis on the female cohort revealed enrichment several pathways in the analyses including symptom variations, with genes associated with transcriptional activities, growth factors, alcoholism pathways, DNA maintenance and targets of *IRF7*, *IRF2* and *AUTS2*. *IRF7* plays an important role in immunity and autoimmunity [65], while *IRF2* is involved in inflammatory and cancer pathogenesis [66]. *AUTS2* is related to neurodevelopment, autism spectrum disorders and intellectual disability [67]. When including response status, we observed processes related to oxidative stress, glutamate receptor binding and other DNA binding functions, as well as neuron projection membrane and axolemma terms. Sirignano et al. also performed gene and pathway analysis in patients undergoing ECT, but found no significant results in their models [9]. Sun et al. performed enrichment analysis in their cohort undergoing ECT and found genes mainly related to synaptic signaling, cell junction organization, axon, presynapse and calcium ion binding [43]. Our significant results add to the scarce literature on this topic, and further studies are necessary to replicate the current findings in larger samples.

Our study has several strengths that highlight the relevance of the findings. It contributes to the limited number of studies analyzing the longitudinal effects of ECT in TRD patients, representing one of the few pieces of evidence supporting potential biological effects of ECT through a longitudinal epigenomic approach. Other strengths of our study include the use of standardized clinical assessments performed before and after ECT treatment. This approach ensures the reliability and comparability of measurements, enabling an accurate longitudinal evaluation of symptoms changes. Additionally, we employed an unbiased epigenome-wide approach and conducted a comprehensive biological longitudinal characterization of DMPs and DMRs.

Also, some limitations should be acknowledged when interpreting the results. One limitation that may be considered is the relatively small sample size. While this factor may limit the generalizability of the findings, it should be viewed in the context of the originality of the study design, as it represents one of the few longitudinal EWAS evaluating the effects of ECT in a cohort of TRD patients. Other considerations should be taken into account regarding the small size. The inherent challenges in performing a longitudinal study with TRD patients treated with ECT treatment should be considered and explain the small sample size. Indeed, to date, few studies performed EWAS analyses in relation to ECT in TRD patients in longitudinal studies on similar sample sizes with a similar duration of treatment. In detail, in the studies performed by Moschny et al. [8] and by and Sirignano et al. [9], 17 and 34 TRD patients, respectively, were treated with ECT for 4/5 weeks. Finally, clinical intervention studies use more homogenous patient groups, reducing the need for very large samples. Despite these limitations, our study with a small cohort may provide valuable information to be replicated in further studies employing multicenter approaches and collaborative efforts. These efforts can increase sample sizes, allowing a more robust characterization of ECT response and providing better insights into the biological processes underlying the effects of ECT. Another limitation is associated with the relevance of peripheral blood methylation to the brain and the biological correspondence between the brain and peripheral tissues. It is important to recognize that methylation differences can vary considerably across different tissues, despite the observed consistent effects of various methylation quantitative trait loci across tissues [68]. Considering this aspect, it is important to consider the difficulties associated with directly assessing brain tissue, as well as the advantages of analyzing peripheral blood samples. This is because epigenetic and transcriptional alterations in peripheral blood, to some extent, reflect the molecular and cellular changes occurring in the brain. Possible effects of anesthesia and pharmacotherapy are potential confounding factors in methylation studies. However, medication in each patient was kept relatively constant during the ECT treatment, and there were no significant differences between patients regarding anesthesia administration. Furthermore, we did not consider potential confounding factors such as gender-related effects, and our analyses were not structured within a control group design. These aspects should be considered and addressed in future studies. Although we did not find significant CpG sites or DMRs after correction for multiple analyses, our results may suggest mechanisms of action of ECT response that need further investigation in larger studies.

## Conclusion

Our study provides valuable insights into the limited available evidence concerning DNA methylation changes associated with ECT treatment. It highlights potential differentially methylated CpG sites DMRs that may play a role in ECT outcomes. The findings point to some genes, regions and pathways involved in the inflammatory and immune system, which is consistent with the inflammatory/immune hypothesis of MDD pathophysiology. Our results may enhance our understanding of the biological mechanisms of action of ECT, as well as its outcomes, in TRD patients.

From a practical clinical perspective, our study may enhance the understanding of the biological underpinnings of ECT’s mechanisms of action and treatment response, allowing for the identification of individuals at higher risk for poor outcomes and the selection of patients who would benefit more from targeted intervention strategies. Better stratification and personalized treatment for patients with TRD, who chronically suffer from the disease, imposing significant individual and socioeconomic burden, are imperative for improved patient care and healthcare management.

In future, well-designed studies may help elucidate additional biomarkers that can predict MDD treatment response and potentially help in treatment options for patients suffering from TRD. The considerable cost and inherent challenges of conducting controlled clinical trials in large samples of patients with TRD, requiring multicenter collaborative efforts, pose difficulties in reproducing findings in larger cohorts. Therefore, results from smaller studies are valuable and should be considered relevant for replication in larger cohorts.

### Supplementary Information

Supplementary material 1.

## Data Availability

Data and materials are available upon request.

## References

[1] Singh A, Kar SK (2017). How electroconvulsive therapy works? Understanding the neurobiological mechanisms. Clin Psychopharmacol Neurosci.

[2] Hermida AP, Glass OM, Shafi H, McDonald WM (2018). Electroconvulsive therapy in depression: current practice and future direction. Psychiatr Clin N Am.

[3] Weiner RD, Reti IM (2017). Key updates in the clinical application of electroconvulsive therapy. Int Rev Psychiatry.

[4] Maffioletti E, Carvalho Silva R, Bortolomasi M, Baune BT, Gennarelli M, Minelli A (2021). Molecular biomarkers of electroconvulsive therapy effects and clinical response: understanding the present to shape the future. Brain Sci.

[5] Kleimann A, Kotsiari A, Sperling W, Gröschl M, Heberlein A, Kahl KG (2015). BDNF serum levels and promoter methylation of BDNF exon I, IV and VI in depressed patients receiving electroconvulsive therapy. J Neural Transm.

[6] Moschny N, Jahn K, Bajbouj M, Maier HB, Ballmaier M, Khan AQ (2020). DNA methylation of the t-PA gene differs between various immune cell subtypes isolated from depressed patients receiving electroconvulsive therapy. Front psychiatry.

[7] Maier HB, Moschny N, Eberle F, Jahn K, Folsche T, Schülke R (2023). DNA methylation of POMC and NR3C1-1F and its implication in major depressive disorder and electroconvulsive therapy. Pharmacopsychiatry.

[8] Moschny N, Zindler T, Jahn K, Dorda M, Davenport CF, Wiehlmann L (2020). Novel candidate genes for ECT response prediction-a pilot study analyzing the DNA methylome of depressed patients receiving electroconvulsive therapy. Clin Epigenet.

[9] Sirignano L, Frank J, Kranaster L, Witt SH, Streit F, Zillich L (2021). Methylome-wide change associated with response to electroconvulsive therapy in depressed patients. Transl Psychiatry.

[10] Thase ME, Rush AJ (1997). When at first you don’t succeed: sequential strategies for antidepressant nonresponders. J Clin Psychiatry.

[11] Minelli A, Zanardini R, Abate M, Bortolomasi M, Gennarelli M, Bocchio-Chiavetto L (2011). Vascular Endothelial Growth Factor (VEGF) serum concentration during electroconvulsive therapy (ECT) in treatment resistant depressed patients. Prog Neuropsychopharmacol Biol Psychiatry.

[12] Montgomery SA, Asberg M (1979). A new depression scale designed to be sensitive to change. Br J Psychiatry.

[13] Pidsley R, Zotenko E, Peters TJ, Lawrence MG, Risbridger GP, Molloy P (2016). Critical evaluation of the Illumina MethylationEPIC BeadChip microarray for whole-genome DNA methylation profiling. Genome Biol.

[14] Du P, Zhang X, Huang C-C, Jafari N, Kibbe WA, Hou L (2010). Comparison of Beta-value and M-value methods for quantifying methylation levels by microarray analysis. BMC Bioinform.

[15] Fortin J-P, Triche TJ, Hansen KD (2017). Preprocessing, normalization and integration of the Illumina HumanMethylationEPIC array with minfi. Bioinformatics.

[16] Zhuang H, Wang H, Ji Z (2022). findPC: an R package to automatically select the number of principal components in single-cell analysis. Bioinformatics.

[17] Sales G, Calura E, Romualdi C (2019). metaGraphite—a new layer of pathway annotation to get metabolite networks. Bioinformatics.

[18] Sales G, Calura E, Cavalieri D, Romualdi C (2012). graphite—a Bioconductor package to convert pathway topology to gene network. BMC Bioinform.

[19] Day-Williams AG, Southam L, Panoutsopoulou K, Rayner NW, Esko T, Estrada K (2011). A variant in MCF2L is associated with osteoarthritis. Am J Hum Genet.

[20] Farrow E, Chiocchetti AG, Rogers JC, Pauli R, Raschle NM, Gonzalez-Madruga K (2021). SLC25A24 gene methylation and gray matter volume in females with and without conduct disorder: an exploratory epigenetic neuroimaging study. Transl Psychiatry.

[21] Kang Y, Zhang K, Sun L, Zhang Y (2022). Regulation and roles of FOXK2 in cancer. Front Oncol.

[22] McClard CK, Kochukov MY, Herman I, Liu Z, Eblimit A, Moayedi Y (2018). POU6f1 mediates neuropeptide-dependent plasticity in the adult brain. J Neurosci.

[23] Xiao W, Geng W, Zhou M, Xu J, Wang S, Huang Q (2022). POU6F1 cooperates with RORA to suppress the proliferation of lung adenocarcinoma by downregulation HIF1A signaling pathway. Cell Death Dis.

[24] Sozio F, Schioppa T, Laffranchi M, Salvi V, Tamassia N, Bianchetto-Aguilera FM (2023). CCRL2 expression by specialized lung capillary endothelial cells controls NK-cell homing in lung cancer. Cancer Immunol Res.

[25] Motwani J, Rodger EJ, Stockwell PA, Baguley BC, Macaulay EC, Eccles MR (2021). Genome-wide DNA methylation and RNA expression differences correlate with invasiveness in melanoma cell lines. Epigenomics.

[26] Zhou Y, Liang Y, Low MJ, Kreek MJ (2019). Nuclear transcriptional changes in hypothalamus of Pomc enhancer knockout mice after excessive alcohol drinking. Genes Brain Behav.

[27] Xu M, Zheng J, Wang J, Huang H, Hu G, He H (2023). MCF2L-AS1/miR-874-3p/STAT3 feedback loop contributes to lung adenocarcinoma cell growth and cisplatin resistance. Heliyon.

[28] Huang S-C, Chen Y-M, Hu Y-Y, Shi Y-J, Xiao Q-W, Li Z (2022). Downregulation of MCF2L promoted the ferroptosis of hepatocellular carcinoma cells through PI3K/mTOR pathway in a RhoA/Rac1 dependent manner. Dis Mark.

[29] Boraska V, Franklin CS, Floyd JAB, Thornton LM, Huckins LM, Southam L (2014). A genome-wide association study of anorexia nervosa. Mol Psychiatry.

[30] Kuang Y, Wang Y, Cao X, Peng C, Gao H (2021). New prognostic factors and scoring system for patients with acute myeloid leukemia. Oncol Lett.

[31] Adanty C, Qian J, Al-Chalabi N, Fatemi AB, Gerretsen P, Graff A (2022). Sex differences in schizophrenia: a longitudinal methylome analysis. J Neural Transm.

[32] Aspra Q, Cabrera-Mendoza B, Morales-Marín ME, Márquez C, Chicalote C, Ballesteros A (2022). Epigenome-wide analysis reveals DNA methylation alteration in ZFP57 and its target RASGFR2 in a Mexican population cohort with autism. Child (Basel, Switzerland).

[33] Rutten BPF, Vermetten E, Vinkers CH, Ursini G, Daskalakis NP, Pishva E (2018). Longitudinal analyses of the DNA methylome in deployed military servicemen identify susceptibility loci for post-traumatic stress disorder. Mol Psychiatry.

[34] Okazaki S, Boku S, Otsuka I, Mouri K, Aoyama S, Shiroiwa K (2016). The cell cycle-related genes as biomarkers for schizophrenia. Prog Neuropsychopharmacol Biol Psychiatry.

[35] Liu X, Malenfant P, Reesor C, Lee A, Hudson ML, Harvard C (2011). 2p15-p16.1 microdeletion syndrome: molecular characterization and association of the OTX1 and XPO1 genes with autism spectrum disorders. Eur J Hum Genet.

[36] Zheng D, Kern L, Elinav E (2021). The NLRP6 inflammasome. Immunology.

[37] Yu Y, Wang Z, Zheng Q, Li J (2021). GALNT2/14 overexpression correlate with prognosis and methylation: potential therapeutic targets for lung adenocarcinoma. Gene.

[38] Carleial S, Nätt D, Unternährer E, Elbert T, Robjant K, Wilker S (2021). DNA methylation changes following narrative exposure therapy in a randomized controlled trial with female former child soldiers. Sci Rep.

[39] Hashimoto Y, Tsuji O, Kanekura K, Aiso S, Niikura T, Matsuoka M (2004). The Gtx homeodomain transcription factor exerts neuroprotection using its homeodomain. J Biol Chem.

[40] Zhang Y-Y, Bao H-L, Dong L-X, Liu Y, Zhang G-W, An F-M (2021). Silenced lncRNA H19 and up-regulated microRNA-129 accelerates viability and restrains apoptosis of PC12 cells induced by Aβ25-35 in a cellular model of Alzheimer’s disease. Cell Cycle.

[41] Karanović J, Ivković M, Jovanović VM, Šviković S, Pantović-Stefanović M, Brkušanin M (2017). Effect of childhood general traumas on suicide attempt depends on TPH2 and ADARB1 variants in psychiatric patients. J Neural Transm.

[42] Simmons M, Meador-Woodruff JH, Sodhi MS (2010). Increased cortical expression of an RNA editing enzyme occurs in major depressive suicide victims. NeuroReport.

[43] Sun H, Bai T, Zhang X, Fan X, Zhang K, Zhang J, et al. Molecular mechanisms underlying structural plasticity of electroconvulsive therapy in major depressive disorder. Brain Imaging Behav. 2024. 10.1007/s11682-024-00884-9 38664360

[44] Van Assche E, Hohoff C, Zang J, Knight MJ, Baune BT (2023). Epigenetic modification related to cognitive changes during a cognitive training intervention in depression. Prog Neuropsychopharmacol Biol Psychiatry.

[45] Yang R, Xu C, Bierer LM, Flory JD, Gautam A, Bader HN (2021). Longitudinal genome-wide methylation study of PTSD treatment using prolonged exposure and hydrocortisone. Transl Psychiatry.

[46] Nanamiya T, Takane K, Yamaguchi K, Okawara Y, Arakawa M, Saku A (2024). Expression of PVRL4, a molecular target for cancer treatment, is transcriptionally regulated by FOS. Oncol Rep.

[47] Liu X, Zhan Y, Xu W, Liu X, Geng Y, Liu L (2021). Prognostic and immunological role of Fam20C in pan-cancer. Biosci Rep.

[48] Yang X, Yu D, Ren Y, Wei J, Pan W, Zhou C (2016). Integrative functional genomics implicates EPB41 dysregulation in hepatocellular carcinoma risk. Am J Hum Genet.

[49] Yuan J, Xing H, Li Y, Song Y, Zhang N, Xie M (2021). EPB41 suppresses the Wnt/β-catenin signaling in non-small cell lung cancer by sponging ALDOC. Cancer Lett.

[50] Liao Y, Yang M, Wang K, Wang Y, Zhong B, Jiang N (2022). Deubiquitinating enzyme OTUB1 in immunity and cancer: good player or bad actor?. Cancer Lett.

[51] Chuang LSH, Matsuo J, Douchi D, Bte Mawan NA, Ito Y (2023). RUNX3 in stem cell and cancer biology. Cells.

[52] Shen J, Liang C, Su X, Wang Q, Ke Y, Fang J (2022). Dysfunction and ceRNA network of the tumor suppressor miR-637 in cancer development and prognosis. Biomark Res.

[53] van Buel EM, Patas K, Peters M, Bosker FJ, Eisel ULM, Klein HC (2015). Immune and neurotrophin stimulation by electroconvulsive therapy: is some inflammation needed after all?. Transl Psychiatry.

[54] Mindt S, Neumaier M, Hoyer C, Sartorius A, Kranaster L (2020). Cytokine-mediated cellular immune activation in electroconvulsive therapy: a CSF study in patients with treatment-resistant depression. World J Biol Psychiatry.

[55] Carvalho Silva R, Pisanu C, Maffioletti E, Menesello V, Bortolomasi M, PROMPT consortium, et al. Biological markers of sex-based differences in major depressive disorder and in antidepressant response. Eur Neuropsychopharmacol. 2023;76:89–107. 10.1016/j.euroneuro.2023.07.012 37595325

[56] Mukherjee S, Guha S, Ikeda M, Iwata N, Malhotra AK, Pe’er I, et al. Excess of homozygosity in the major histocompatibility complex in schizophrenia. Hum Mol Genet. 2014;23(22):6088–95 10.1093/hmg/ddu308 PMC4204767 24943592

[57] Zhang Z, Zheng F, You Y, Ma Y, Lu T, Yue W (2016). Growth arrest specific gene 7 is associated with schizophrenia and regulates neuronal migration and morphogenesis. Mol Brain.

[58] Ma H, Su L, Xia W, Wang W, Tan G, Jiao J (2021). MacroH2A1.2 deficiency leads to neural stem cell differentiation defects and autism-like behaviors. EMBO Rep.

[59] Fraporti TT, Contini V, Tovo-Rodrigues L, Recamonde-Mendoza M, Rovaris DL, Rohde LA (2019). Synergistic effects between ADORA2A and DRD2 genes on anxiety disorders in children with ADHD. Prog Neuropsychopharmacol Biol Psychiatry.

[60] Marini C, Hardies K, Pisano T, May P, Weckhuysen S, Cellini E (2017). Recessive mutations in SLC35A3 cause early onset epileptic encephalopathy with skeletal defects. Am J Med Genet A.

[61] Meng S-X, Wang B, Li W-T (2020). Serum expression of EAAT2 and ADORA2A in patients with different degrees of Alzheimer’s disease. Eur Rev Med Pharmacol Sci.

[62] Lin PB-C, Tsai AP-Y, Soni D, Lee-Gosselin A, Moutinho M, Puntambekar SS, et al. INPP5D deficiency attenuates amyloid pathology in a mouse model of Alzheimer’s disease. Alzheimers Dement. 2023;19(6):2528–37. 10.1002/alz.12849 36524682

[63] Pariante CM (2017). Why are depressed patients inflamed? A reflection on 20 years of research on depression, glucocorticoid resistance and inflammation. Eur Neuropsychopharmacol.

[64] Lo Iacono L, Bussone S, Andolina D, Tambelli R, Troisi A, Carola V (2020). Dissecting major depression: the role of blood biomarkers and adverse childhood experiences in distinguishing clinical subgroups. J Affect Disord.

[65] Ma W, Huang G, Wang Z, Wang L, Gao Q (2023). IRF7: role and regulation in immunity and autoimmunity. Front Immunol.

[66] Liao W, Overman MJ, Boutin AT, Shang X, Zhao D, Dey P (2019). KRAS-IRF2 axis drives immune suppression and immune therapy resistance in colorectal cancer. Cancer Cell.

[67] Biel A, Castanza AS, Rutherford R, Fair SR, Chifamba L, Wester JC (2022). AUTS2 syndrome: molecular mechanisms and model systems. Front Mol Neurosci.

[68] Liew C-C, Ma J, Tang H-C, Zheng R, Dempsey AA (2006). The peripheral blood transcriptome dynamically reflects system wide biology: a potential diagnostic tool. J Lab Clin Med.

