# Supplementary material for: Unraveling epigenomic signatures and effectiveness of electroconvulsive therapy in treatment-resistant depression patients: a prospective longitudinal study
Source: Clin Epigenetics. 2024 Jul 17;16:93. doi: 10.1186/s13148-024-01704-z (PMC11256624; doi:10.1186/s13148-024-01704-z)
Supplement: Supplementary file 1 — Supplementary material 1. [file 13148_2024_1704_MOESM1_ESM.docx]

**SUPPLEMENTARY MATERIAL**

**Table 1S:** Differentially methylated CpG sites obtained with longitudinal analyses conducted through T0-T1 timepoint with a nominal p-value ≤ 10^-5^. The Table shows a) the differentially methylated probes (DMPs) at T0-T1 and the DMPs analysed with covariates including b) clinical symptoms variations (assessed by delta MADRS), c) response (reduction greater than 50% in MADRS score at T1). The results are ordered by the nominal p-values. MADRS: Montgomery-Åsberg Depression Rating Scale

| **a) T0-T1** | | | | | |
| --- | --- | --- | --- | --- | --- |
| CpG | CHR | Base pair position | p-value | FDR (q) | Annotated gene |
| cg24392473 | 16 | 69497645 | 1.44E-07 | 0.1044 | *CYB5B* |
| cg22395624 | 4 | 6735531 | 5.89E-06 | 1.0000 |  |
| cg08158262 | 1 | 161054019 | 7.62E-06 | 1.0000 | *PVRL4* |
| **b) T0-T1 (Delta MADRS)** | | | | | |
| CpG | CHR | Base pair position | p-value | FDR (q) | Annotated gene |
| cg14727550 | 7 | 228114 | 5.20E-06 | 1.0000 | *FAM20C* |
| cg03429968 | 1 | 29245850 | 6.42E-06 | 1.0000 | *EPB41* |
| cg20093992 | 11 | 63752267 | 6.89E-06 | 1.0000 | *OTUB1* |
| cg08342736 | 21 | 46631146 | 9.16E-06 | 1.0000 | *ADARB1* |
| **c) T0-T1 (Response status)** | | | | | |
| CpG | CHR | Base pair position | p-value | FDR (q) | Annotated gene |
| cg08237545 | 12 | 28369461 | 2.12E-06 | 1.0000 |  |
| cg23454205 | 7 | 2649748 | 8.24E-06 | 1.0000 | *IQCE* |
| cg14727550 | 7 | 228114 | 8.51E-06 | 1.0000 | *FAM20C* |

**Table 2S.** Regional analysis showing differentially methylated regions (DMRs) in correlational analyses performed in T0-T1. The Table shows a) the DMRs at T0-T1 and the DMRs analysed with covariates including b) clinical symptoms variations (assessed by delta MADRS), and c) response (reduction greater than 50% in MADRS score at T1). Results show DMRs with q values (adjusted after false discovery rate correction) ≤ 0.1 and are ordered by the nominal p-values. MADRS: Montgomery-Åsberg Depression Rating Scale.

| **a) T0-T1** | | | | | | | | |
| --- | --- | --- | --- | --- | --- | --- | --- | --- |
|  | CHR | Start | End | p-value | FDR (q) | p-value area | Adjusted p-value area | Main genes |
| 1 | chr13 | 113698409 | 113699016 | 0.00E+00 | 0.0000 | 0.0001 | 0.0524 | *MCF2L* |
| 2 | chr1 | 108743479 | 108743879 | 3.99E-06 | 0.0028 | 0.0002 | 0.0524 | *SLC25A24* |
| 3 | chr1 | 25228583 | 25228934 | 7.99E-06 | 0.0033 | 0.0002 | 0.0524 | *RUNX3* |
| 4 | chr19 | 3961363 | 3961765 | 1.20E-05 | 0.0033 | 0.0002 | 0.0524 | *MIR637* |
| 5 | chr17 | 80545021 | 80545454 | 1.20E-05 | 0.0033 | 0.0002 | 0.0524 | *FOXK2* |
| 6 | chr11 | 47607761 | 47608722 | 2.00E-05 | 0.0046 | 0.0002 | 0.0524 | *FAM180B* |
| 7 | chr12 | 51592060 | 51592432 | 4.79E-05 | 0.0094 | 0.0005 | 0.0870 | *POU6F1* |
| 8 | chr3 | 46718942 | 46719625 | 5.99E-05 | 0.0103 | 0.0005 | 0.0870 | *ALS2CL* |
| 9 | chr3 | 46448869 | 46449313 | 1.40E-04 | 0.0214 | 0.0006 | 0.0870 | *CCRL2* |
| 10 | chr3 | 118864953 | 118865255 | 1.80E-04 | 0.0248 | 0.0008 | 0.1036 | *TEX55* |
| 11 | chr6 | 31860645 | 31860763 | 3.08E-04 | 0.0354 | 0.0012 | 0.1236 | *EHMT2* |
| 12 | chr19 | 15236793 | 15236852 | 3.08E-04 | 0.0354 | 0.0012 | 0.1236 | *ILVBL* |
| 14 | chr20 | 2636147 | 2636597 | 3.59E-04 | 0.0360 | 0.0013 | 0.1236 | *SNORA51* |
| 13 | chr19 | 11289350 | 11289376 | 3.71E-04 | 0.0360 | 0.0019 | 0.1364 | *KANK2* |
| 15 | chr2 | 241513651 | 241514065 | 3.91E-04 | 0.0360 | 0.0012 | 0.1236 | *RNPEPL1* |
| 16 | chr11 | 9113153 | 9113194 | 4.59E-04 | 0.0374 | 0.0015 | 0.1268 | *SCUBE2* |
| 17 | chr17 | 46871583 | 46871905 | 4.79E-04 | 0.0374 | 0.0016 | 0.1268 | *TTLL6* |
| 19 | chr17 | 19976570 | 19977156 | 4.87E-04 | 0.0374 | 0.0015 | 0.1268 | *SPECC1* |
| 18 | chr5 | 131395684 | 131395888 | 5.23E-04 | 0.0380 | 0.0023 | 0.1364 | *IL3* |
| 20 | chr7 | 107643594 | 107643713 | 6.15E-04 | 0.0390 | 0.0025 | 0.1364 | *LAMB1* |
| 21 | chr14 | 37051874 | 37052039 | 6.15E-04 | 0.0390 | 0.0025 | 0.1364 | *NKX2-8* |
| 24 | chr2 | 30454893 | 30455114 | 6.47E-04 | 0.0390 | 0.0017 | 0.1298 | *LBH* |
| 22 | chr8 | 38964298 | 38964578 | 6.75E-04 | 0.0390 | 0.0026 | 0.1364 | *ADAM32* |
| 23 | chr10 | 134790770 | 134791043 | 6.79E-04 | 0.0390 | 0.0026 | 0.1364 | *LINC01168* |
| 25 | chr10 | 712033 | 712592 | 9.27E-04 | 0.0511 | 0.0021 | 0.1364 | *DIP2C-AS1* |
| 26 | chr3 | 27525880 | 27525996 | 1.08E-03 | 0.0569 | 0.0033 | 0.1364 | *SLC4A7* |
| 27 | chr3 | 114102566 | 114102615 | 1.14E-03 | 0.0569 | 0.0034 | 0.1364 | *ZBTB20* |
| 28 | chr19 | 5784793 | 5784947 | 1.15E-03 | 0.0569 | 0.0034 | 0.1364 | *PRR22* |
| 29 | chr14 | 20903960 | 20904320 | 1.20E-03 | 0.0570 | 0.0025 | 0.1364 | *KLHL33* |
| 30 | chr3 | 171758174 | 171758210 | 1.29E-03 | 0.0592 | 0.0027 | 0.1364 | *FNDC3B* |
| 31 | chr6 | 30878400 | 30878530 | 1.33E-03 | 0.0592 | 0.0028 | 0.1364 | *GTF2H4* |
| 32 | chr2 | 54483478 | 54483817 | 1.42E-03 | 0.0601 | 0.0038 | 0.1384 | *TSPYL6* |
| 33 | chr6 | 33248249 | 33248629 | 1.46E-03 | 0.0601 | 0.0030 | 0.1364 | *B3GALT4* |
| 34 | chr6 | 30165962 | 30166173 | 1.48E-03 | 0.0601 | 0.0031 | 0.1364 | *TRIM26* |
| 35 | chr13 | 107028858 | 107029123 | 1.60E-03 | 0.0619 | 0.0033 | 0.1364 | *LINC00460* |
| 36 | chr17 | 48546342 | 48546620 | 1.67E-03 | 0.0619 | 0.0035 | 0.1364 | *CHAD* |
| 37 | chr20 | 56287005 | 56287532 | 1.69E-03 | 0.0619 | 0.0036 | 0.1364 | *PMEPA1* |
| 39 | chr17 | 78939980 | 78940161 | 1.75E-03 | 0.0619 | 0.0038 | 0.1384 | *RPTOR* |
| 38 | chr12 | 133757352 | 133757594 | 1.78E-03 | 0.0619 | 0.0042 | 0.1504 | *ZNF268* |
| 40 | chr3 | 127317575 | 127317798 | 1.79E-03 | 0.0619 | 0.0242 | 0.3174 | *MCM2* |
| 41 | chr16 | 89972594 | 89972864 | 1.92E-03 | 0.0648 | 0.0074 | 0.2095 | *MC1R* |
| 43 | chr13 | 107570443 | 107570511 | 2.08E-03 | 0.0677 | 0.0261 | 0.3302 | *LINC00443* |
| 42 | chr8 | 10469311 | 10469596 | 2.11E-03 | 0.0677 | 0.0046 | 0.1591 | *RP1L1* |
| 45 | chr2 | 131850876 | 131850909 | 2.36E-03 | 0.0740 | 0.0279 | 0.3424 | *FAM168B* |
| 44 | chr1 | 1114964 | 1115083 | 2.47E-03 | 0.0757 | 0.0050 | 0.1690 | *TTLL10* |
| 46 | chr1 | 207277451 | 207277581 | 2.71E-03 | 0.0813 | 0.0053 | 0.1748 | *C4BPA* |
| 52 | chr7 | 135412850 | 135412849 | 3.00E-03 | 0.0881 | 0.1913 | 0.6173 | *SLC13A4* |
| 47 | chr6 | 31850190 | 31850515 | 3.07E-03 | 0.0883 | 0.0058 | 0.1865 | *EHMT2* |
| 48 | chr2 | 69002175 | 69002378 | 3.33E-03 | 0.0910 | 0.0061 | 0.1916 | *ARHGAP25* |
| 55 | chr10 | 134145730 | 134145729 | 3.36E-03 | 0.0910 | 0.1974 | 0.6173 | *LRRC27* |
| 49 | chr14 | 81408276 | 81408461 | 3.36E-03 | 0.0910 | 0.0332 | 0.3673 | *CEP128* |
| 50 | chr8 | 86133289 | 86133291 | 3.77E-03 | 0.0967 | 0.0349 | 0.3701 | *CA13* |
| 51 | chr14 | 34932263 | 34932264 | 3.78E-03 | 0.0967 | 0.0350 | 0.3701 | *SPTSSA* |
| 53 | chr8 | 49532876 | 49533187 | 3.85E-03 | 0.0967 | 0.0068 | 0.2039 | *EFCAB1* |
| 54 | chr6 | 30509367 | 30509467 | 3.86E-03 | 0.0967 | 0.0068 | 0.2039 | *GNL1* |
| 56 | chr6 | 30878865 | 30879000 | 3.93E-03 | 0.0967 | 0.0069 | 0.2039 | *VARS2* |
| **b) T0-T1 (Delta MADRS)** | | | | | | | | |
|  | CHR | Start | End | p-value | FDR (q) | p-value area | Adjusted p-value area | Main genes |
| 1 | chr20 | 61915242 | 61915663 | 1,06E-04 | 0.0272 | 0.0006 | 0.1283 | *ARFGAP1* |
| 2 | chr20 | 33104053 | 33104234 | 1,10E-04 | 0.0272 | 0.0004 | 0.1283 | *DYNLRB1* |
| 3 | chr14 | 101201612 | 101201649 | 1,14E-04 | 0.0272 | 0.0001 | 0.1283 | *DLK1* |
| 4 | chr16 | 28565113 | 28565427 | 1,18E-04 | 0.0272 | 0.0005 | 0.1283 | *SGF29* |
| 5 | chr5 | 140743576 | 140744076 | 1,35E-04 | 0.0272 | 0.0005 | 0.1283 | *PCDHGA5* |
| 6 | chr9 | 136325050 | 136325323 | 2,41E-04 | 0.0363 | 0.0013 | 0.1371 | *CACFD1* |
| 7 | chr16 | 2301627 | 2301887 | 2,62E-04 | 0.0363 | 0.0008 | 0.1317 | *ECI1* |
| 8 | chr7 | 124570048 | 124570359 | 3,26E-04 | 0.0363 | 0.0015 | 0.1371 | *POT1* |
| 9 | chr8 | 29952656 | 29953066 | 3,26E-04 | 0.0363 | 0.0010 | 0.1371 | *LEPROTL1* |
| 10 | chr2 | 232063177 | 232063399 | 4,99E-04 | 0.0500 | 0.0013 | 0.1371 | *ARMC9* |
| 12 | chr19 | 39522945 | 39522944 | 5,84E-04 | 0.0500 | 0.0846 | 0.6169 | *FBXO27* |
| 13 | chr11 | 82612023 | 82612022 | 6,30E-04 | 0.0500 | 0.0930 | 0.6264 | *PRCP* |
| 11 | chr14 | 53173498 | 53173770 | 6,47E-04 | 0.0500 | 0.0015 | 0.1371 | *PSMC6* |
| 14 | chr1 | 230415344 | 230415902 | 7,23E-04 | 0.0519 | 0.0022 | 0.1851 | *GALNT2* |
| 16 | chr5 | 138940695 | 138940959 | 9,64E-04 | 0.0617 | 0.0042 | 0.2350 | *UBE2D2* |
| 15 | chr17 | 45908475 | 45908635 | 1,04E-03 | 0.0617 | 0.0086 | 0.3454 | *MRPL10* |
| 17 | chr16 | 2034369 | 2034545 | 1,04E-03 | 0.0617 | 0.0026 | 0.2039 | *GFER* |
| 19 | chr8 | 27631774 | 27631987 | 1,33E-03 | 0.0743 | 0.0030 | 0.2175 | *ESCO2* |
| 18 | chr6 | 111195302 | 111195554 | 1,45E-03 | 0.0764 | 0.0099 | 0.3533 | *AMD1* |
| 20 | chr20 | 42939768 | 42939820 | 1,60E-03 | 0.0790 | 0.0310 | 0.5137 | *FITM2* |
| 21 | chr20 | 2632525 | 2632718 | 1,66E-03 | 0.0790 | 0.0035 | 0.2261 | *NOP56* |
| 22 | chr10 | 35379231 | 35379348 | 1,73E-03 | 0.0790 | 0.0036 | 0.2261 | *CUL2* |
| 24 | chr1 | 27216733 | 27216793 | 2,22E-03 | 0.0971 | 0.0042 | 0.2350 | *GPN2* |
| **c) T0-T1 (Response status)** | | | | | | | | |
|  | CHR | Start | End | p-value | FDR (q) | p-value area | Adjusted p-value area | Main genes |
| 1 | chr19 | 49993866 | 49994336 | 1.24E-05 | 0.0093 | 0.0002 | 0.0754 | *SNORD34* |
| 2 | chr11 | 278369 | 278538 | 2.49E-05 | 0.0093 | 0.0003 | 0.0754 | *NLRP6* |
| 4 | chr1 | 230415226 | 230416101 | 2.90E-05 | 0.0093 | 0.0003 | 0.0754 | *GALNT2* |
| 3 | chr2 | 128458241 | 128458556 | 3.32E-05 | 0.0093 | 0.0002 | 0.0754 | *SFT2D3* |
| 5 | chr20 | 61915242 | 61915663 | 9.13E-05 | 0.0204 | 0.0005 | 0.1067 | *ARFGAP1* |
| 6 | chr14 | 106321937 | 106322429 | 2.90E-04 | 0.0517 | 0.0013 | 0.1587 | *FAM30A* |
| 7 | chr1 | 155100383 | 155100511 | 3.98E-04 | 0.0517 | 0.0024 | 0.1928 | *EFNA1* |
| 8 | chr17 | 61819249 | 61819520 | 4.48E-04 | 0.0517 | 0.0011 | 0.1587 | *STRADA* |
| 9 | chr2 | 70056603 | 70056697 | 4.90E-04 | 0.0517 | 0.0012 | 0.1587 | *GMCL1* |
| 10 | chr19 | 38747080 | 38747292 | 5.39E-04 | 0.0517 | 0.0013 | 0.1587 | *PPP1R14A* |
| 11 | chr2 | 56410012 | 56410143 | 5.77E-04 | 0.0517 | 0.0018 | 0.1928 | *CCDC85A* |
| 12 | chr12 | 118810864 | 118811074 | 5.85E-04 | 0.0517 | 0.0029 | 0.1935 | *TAOK3* |
| 13 | chr19 | 11670208 | 11670275 | 6.02E-04 | 0.0517 | 0.0029 | 0.1935 | *ELOF1* |
| 15 | chr12 | 52463649 | 52464244 | 8.05E-04 | 0.0608 | 0.0019 | 0.1928 | *ATG101* |
| 14 | chr17 | 45908475 | 45908635 | 8.55E-04 | 0.0608 | 0.0071 | 0.2938 | *MRPL10* |
| 17 | chr6 | 31540400 | 31540750 | 9.04E-04 | 0.0608 | 0.0024 | 0.1928 | *LTA* |
| 16 | chr12 | 131356527 | 131356597 | 9.25E-04 | 0.0608 | 0.0023 | 0.1928 | *RAN* |
| 18 | chr11 | 64120624 | 64120685 | 1.24E-03 | 0.0771 | 0.0087 | 0.3031 | *CCDC88B* |
| 19 | chr8 | 125551213 | 125551332 | 1.31E-03 | 0.0771 | 0.0029 | 0.1935 | *TATDN1* |
| 20 | chr7 | 12443147 | 12443529 | 1.55E-03 | 0.0855 | 0.0047 | 0.2747 | *VWDE* |
| 21 | chr12 | 109592935 | 109592987 | 1.61E-03 | 0.0855 | 0.0290 | 0.4559 | *ACACB* |
| 23 | chr3 | 183415828 | 183416068 | 1.70E-03 | 0.0864 | 0.0297 | 0.4559 | *YEATS2* |
| 22 | chr16 | 15596083 | 15596423 | 1.99E-03 | 0.0920 | 0.0054 | 0.2899 | *BMERB1* |
| 25 | chr20 | 33291893 | 33291894 | 2.00E-03 | 0.0920 | 0.0322 | 0.4559 | *TP53INP2* |
| 24 | chr15 | 83478289 | 83478411 | 2.06E-03 | 0.0920 | 0.0055 | 0.2899 | *WHAMM* |
| 26 | chr8 | 29953065 | 29953601 | 2.22E-03 | 0.0954 | 0.0043 | 0.2680 | *LEPROTL1* |

**Table 3S:** Analyses performed on the female cohort. Differentially methylated CpG sites obtained with longitudinal analyses conducted through T0-T1 timepoints, with a nominal p-value ≤ 10^-5^. The Table shows a) the differentially methylated probes (DMPs) at T0-T1 and the DMPs analysed with covariates including b) clinical symptoms variations (assessed by delta MADRS), c) response (reduction greater than 50% in MADRS score at T1). The results are ordered by the nominal p-values. MADRS: Montgomery-Åsberg Depression Rating Scale

| **a) T0-T1** | | | | | |
| --- | --- | --- | --- | --- | --- |
| CpG | CHR | Base pair position | p-value | FDR (q) | Annotated gene |
| cg15464763 | 7 | 148497944 | 3.27E-06 | 1.0000 | *CUL1* |
| cg07361264 | 7 | 4772405 | 5.20E-06 | 1.0000 | *FOXK1* |
| cg05149226 | 12 | 126265319 | 6.90E-06 | 1.0000 |  |
| cg24392473 | 16 | 69497645 | 8.29E-06 | 1.0000 | *CYB5B* |
| **b) T0-T1 (Delta MADRS)** | | | | | |
| CpG | CHR | Base pair position | p-value | FDR (q) | Annotated gene |
| cg06810819 | 8 | 117768886 | 1.11E-06 | 0.6557 | *EIF3H* |
| cg24158620 | 16 | 71741975 | 2.48E-06 | 0.6557 | *PHLPP2* |
| cg22343220 | 9 | 129992496 | 2.71E-06 | 0.6557 |  |
| cg14727550 | 7 | 228114 | 3.88E-06 | 0.6851 | *FAM20C* |
| cg04352342 | 8 | 67024748 | 4.73E-06 | 0.6851 |  |
| cg09249877 | 7 | 111731013 | 6.31E-06 | 0.7252 | *DOCK4* |
| cg24954648 | 15 | 77220591 | 8.22E-06 | 0.7252 |  |
| **c) T0-T1 (Response status)** | | | | | |
| CpG | CHR | Base pair position | p-value | FDR (q) | Annotated gene |
| cg06252355 | 3 | 195477915 | 1.68E-06 | 0.6650 | *MUC4* |
| cg01616161 | 9 | 139345326 | 1.93E-06 | 0.6650 | *SEC16A* |
| cg05492975 | 20 | 60119508 | 2.75E-06 | 0.6650 | *CDH4* |
| cg24954648 | 15 | 77220591 | 7.24E-06 | 1.0000 |  |
| cg10881098 | 3 | 129152439 | 8.17E-06 | 1.0000 | *MBD4* |
| cg06546571 | 10 | 34526765 | 8.41E-06 | 1.0000 | *PARD3* |

**Table 4S.** Analyses performed on the female cohort. Regional analysis showing differentially methylated regions (DMRs) in correlational analyses performed in T0-T1. The Table shows a) the DMRs at T0-T1 and the DMRs analysed with covariates including b) clinical symptoms variations (assessed by delta MADRS) and c) response (reduction greater than 50% in MADRS score at T1). Results show DMRs with q values (adjusted after false discovery rate correction) ≤ 0.1 and are ordered by the nominal p-values. Results with an adjusted p-value area of ≤ 0.05 are highlighted in bold. MADRS: Montgomery-Åsberg Depression Rating Scale.

| **a) T0-T1** | | | | | | | | |
| --- | --- | --- | --- | --- | --- | --- | --- | --- |
|  | CHR | Start | End | p-value | FDR (q) | p-value area | Adjusted p-value area | Main genes |
| 1 | chr19 | 3961363 | 3961765 | 0.00E+00 | 0.0000 | 0.0001 | 0.0781 | *MIR637* |
| 2 | chr10 | 99446789 | 99447166 | 4.00E-06 | 0.0026 | 0.0002 | 0.0781 | *AVPI1* |
| 3 | chr1 | 25228583 | 25228922 | 8.00E-06 | 0.0034 | 0.0003 | 0.0781 | *RUNX3* |
| 4 | chr13 | 113698409 | 113698688 | 1.20E-05 | 0.0039 | 0.0003 | 0.0781 | *MCF2L* |
| 5 | chr17 | 80545021 | 80545454 | 2.00E-05 | 0.0051 | 0.0003 | 0.0781 | *FOXK2* |
| 6 | chr2 | 225266784 | 225267119 | 3.60E-05 | 0.0077 | 0.0004 | 0.0781 | *FAM124B* |
| 7 | chr12 | 51592060 | 51592432 | 6.80E-05 | 0.0125 | 0.0004 | 0.0824 | *POU6F1* |
| 8 | chr3 | 16646376 | 16646582 | 1.52E-04 | 0.0245 | 0.0013 | 0.1330 | *DAZL* |
| 9 | chr5 | 131395684 | 131395888 | 1.72E-04 | 0.0246 | 0.0014 | 0.1330 | *IL3* |
| 10 | chr17 | 19976821 | 19977156 | 2.72E-04 | 0.0350 | 0.0016 | 0.1330 | *SPECC1* |
| 11 | chr6 | 31740806 | 31740978 | 4.12E-04 | 0.0368 | 0.0011 | 0.1330 | *VWA7* |
| 13 | chr5 | 92918518 | 92918560 | 4.20E-04 | 0.0368 | 0.0060 | 0.1824 | *NR2F1* |
| 12 | chr5 | 154320552 | 154320902 | 4.32E-04 | 0.0368 | 0.0012 | 0.1330 | *MRPL22* |
| 15 | chr19 | 17858608 | 17858829 | 4.68E-04 | 0.0368 | 0.0031 | 0.1580 | *FCHO1* |
| 14 | chr19 | 45448960 | 45449165 | 4.76E-04 | 0.0368 | 0.0015 | 0.1330 | *APOC2* |
| 18 | chr10 | 111767585 | 111767867 | 5.04E-04 | 0.0368 | 0.0016 | 0.1330 | *ADD3* |
| 16 | chr19 | 56057009 | 56057386 | 5.08E-04 | 0.0368 | 0.0019 | 0.1330 | *SBK3* |
| 17 | chr7 | 95114681 | 95115036 | 5.28E-04 | 0.0368 | 0.0022 | 0.1477 | *ASB4* |
| 19 | chr14 | 74485814 | 74485855 | 5.76E-04 | 0.0368 | 0.0035 | 0.1649 | *ENTPD5* |
| 20 | chr10 | 62332353 | 62332453 | 5.96E-04 | 0.0368 | 0.0017 | 0.1330 | *ANK3* |
| 21 | chr17 | 46871795 | 46872075 | 6.00E-04 | 0.0368 | 0.0017 | 0.1330 | *TTLL6* |
| 23 | chr10 | 131812412 | 131812878 | 6.72E-04 | 0.0385 | 0.0018 | 0.1330 | *EBF3* |
| 22 | chr11 | 65374664 | 65374833 | 6.88E-04 | 0.0385 | 0.0040 | 0.1649 | *MAP3K11* |
| 24 | chr8 | 38964298 | 38964578 | 7.68E-04 | 0.0408 | 0.0026 | 0.1533 | *ADAM32* |
| 25 | chr7 | 96652223 | 96652481 | 7.92E-04 | 0.0408 | 0.0026 | 0.1533 | *DLX5* |
| 26 | chr20 | 48729305 | 48729561 | 9.32E-04 | 0.0461 | 0.0147 | 0.3003 | *UBE2V1* |
| 27 | chr20 | 2636392 | 2636597 | 1.03E-03 | 0.0492 | 0.0030 | 0.1580 | *SNORA51* |
| 28 | chr5 | 38468218 | 38468525 | 1.09E-03 | 0.0502 | 0.0027 | 0.1533 | *EGFLAM* |
| 29 | chr3 | 118864953 | 118865135 | 1.15E-03 | 0.0510 | 0.0027 | 0.1533 | *TEX55* |
| 30 | chr6 | 29395658 | 29395950 | 1.23E-03 | 0.0527 | 0.0056 | 0.1822 | *OR11A1* |
| 31 | chr3 | 114102566 | 114102615 | 1.27E-03 | 0.0528 | 0.0035 | 0.1649 | *ZBTB20* |
| 33 | chr1 | 29213811 | 29214297 | 1.49E-03 | 0.0566 | 0.0038 | 0.1649 | *EPB41* |
| 32 | chr7 | 73082341 | 73082469 | 1.50E-03 | 0.0566 | 0.0064 | 0.1874 | *VPS37D* |
| 34 | chr12 | 8850186 | 8850385 | 1.50E-03 | 0.0566 | 0.0038 | 0.1649 | *RIMKLB* |
| 35 | chr2 | 242169320 | 242169936 | 1.59E-03 | 0.0584 | 0.0038 | 0.1649 | *ANO7* |
| 37 | chr3 | 43020113 | 43020214 | 1.76E-03 | 0.0601 | 0.0070 | 0.1948 | *GASK1A* |
| 36 | chr2 | 10262788 | 10262827 | 1.77E-03 | 0.0601 | 0.0048 | 0.1822 | *RRM2* |
| 38 | chr6 | 30104296 | 30104754 | 1.79E-03 | 0.0601 | 0.0050 | 0.1822 | *TRIM40* |
| 39 | chr21 | 47575135 | 47575134 | 1.82E-03 | 0.0601 | 0.1541 | 0.6447 | *FTCD* |
| 41 | chr16 | 620305 | 620304 | 1.97E-03 | 0.0623 | 0.1592 | 0.6463 | *PIGQ* |
| 42 | chr6 | 33756679 | 33756678 | 1.98E-03 | 0.0623 | 0.1597 | 0.6463 | *LEMD2* |
| 40 | chr12 | 133757352 | 133757594 | 2.11E-03 | 0.0646 | 0.0048 | 0.1822 | *ZNF268* |
| 43 | chr14 | 37051903 | 37052039 | 2.30E-03 | 0.0675 | 0.0081 | 0.2040 | *NKX2-8* |
| 44 | chr6 | 143381344 | 143381675 | 2.31E-03 | 0.0675 | 0.0050 | 0.1822 | *AIG1* |
| 45 | chr19 | 11289350 | 11289376 | 2.50E-03 | 0.0714 | 0.0053 | 0.1822 | *KANK2* |
| 46 | chr22 | 36013676 | 36013708 | 2.74E-03 | 0.0759 | 0.0056 | 0.1822 | *MB* |
| 50 | chr5 | 118604541 | 118604833 | 2.82E-03 | 0.0759 | 0.0091 | 0.2122 | *TNFAIP8* |
| 47 | chr19 | 17212317 | 17212553 | 2.85E-03 | 0.0759 | 0.0057 | 0.1822 | *MYO9B* |
| 48 | chr6 | 33248547 | 33248687 | 2.94E-03 | 0.0759 | 0.0058 | 0.1822 | *B3GALT4* |
| 49 | chr2 | 54483478 | 54483817 | 2.95E-03 | 0.0759 | 0.0058 | 0.1822 | *TSPYL6* |
| 51 | chr10 | 134790770 | 134791043 | 3.43E-03 | 0.0865 | 0.0064 | 0.1874 | *LINC01168* |
| 52 | chr7 | 150554916 | 150555302 | 3.86E-03 | 0.0954 | 0.0070 | 0.1948 | *AOC1* |
| 53 | chr19 | 52643383 | 52643487 | 3.96E-03 | 0.0954 | 0.0072 | 0.1948 | *ZNF616* |
| 55 | chr12 | 107486802 | 107487097 | 4.00E-03 | 0.0954 | 0.0073 | 0.1948 | *CRY1* |
| 56 | chr6 | 30509367 | 30509467 | 4.12E-03 | 0.0964 | 0.0075 | 0.1957 | *GNL1* |
| 57 | chr5 | 43602117 | 43602519 | 4.26E-03 | 0.0978 | 0.0076 | 0.1958 | *NNT* |
| **b) T0-T1 (Delta MADRS)** | | | | | | | | |
|  | CHR | Start | End | p-value | FDR (q) | p-value area | Adjusted p-value area | Main genes |
| **1** | **chr6** | **29648162** | **29649024** | **0.00E+00** | **0.0000** | **0.0000** | **0.0313** | ***ZFP57*** |
| **2** | **chr16** | **28995844** | **28996533** | **0.00E+00** | **0.0000** | **0.0001** | **0.0313** | ***LAT*** |
| **3** | **chr17** | **48049953** | **48050512** | **0.00E+00** | **0.0000** | **0.0001** | **0.0313** | ***DLX4*** |
| **4** | **chr7** | **124569714** | **124570359** | **0.00E+00** | **0.0000** | **0.0001** | **0.0313** | ***POT1*** |
| **5** | **chr11** | **67121100** | **67121358** | **1.65E-05** | **0.0036** | **0.0002** | **0.0448** | ***POLD4*** |
| 6 | chr5 | 54518746 | 54519686 | 9.92E-05 | 0.0179 | 0.0005 | 0.0844 | *MCIDAS* |
| 7 | chr2 | 63285098 | 63285739 | 1.45E-04 | 0.0224 | 0.0005 | 0.0844 | *OTX1* |
| 8 | chr2 | 232063177 | 232063399 | 1.78E-04 | 0.0241 | 0.0006 | 0.0873 | *ARMC9* |
| 9 | chr5 | 37834910 | 37835168 | 2.48E-04 | 0.0285 | 0.0008 | 0.0985 | *GDNF* |
| 10 | chr3 | 50387781 | 50388132 | 2.81E-04 | 0.0285 | 0.0009 | 0.0985 | *NPRL2* |
| 12 | chr4 | 183061831 | 183062004 | 2.89E-04 | 0.0285 | 0.0011 | 0.0999 | *FBXO27* |
| 11 | chr19 | 39522945 | 39522944 | 3.18E-04 | 0.0287 | 0.0246 | 0.3861 | *TENM3* |
| 13 | chr22 | 24823390 | 24823554 | 3.85E-04 | 0.0320 | 0.0011 | 0.0999 | *ADORA2A* |
| 14 | chr7 | 30951426 | 30951801 | 4.59E-04 | 0.0335 | 0.0012 | 0.0999 | *AQP1* |
| 15 | chr16 | 3174220 | 3174435 | 5.09E-04 | 0.0335 | 0.0028 | 0.1266 | *ZNF205-AS1* |
| 17 | chr10 | 35379231 | 35379348 | 5.09E-04 | 0.0335 | 0.0016 | 0.1058 | *OSM* |
| 16 | chr22 | 30662973 | 30663316 | 5.33E-04 | 0.0335 | 0.0013 | 0.1027 | *CUL2* |
| 18 | chr15 | 66797304 | 66797614 | 5.62E-04 | 0.0335 | 0.0029 | 0.1266 | *ZWILCH* |
| 20 | chr20 | 2632525 | 2632718 | 6.41E-04 | 0.0335 | 0.0018 | 0.1077 | *CLIC6* |
| 19 | chr21 | 36041528 | 36041699 | 6.45E-04 | 0.0335 | 0.0016 | 0.1058 | *NOP56* |
| 21 | chr6 | 137243220 | 137243669 | 6.49E-04 | 0.0335 | 0.0018 | 0.1077 | *SLC35D3* |
| 25 | chr12 | 14927805 | 14928083 | 8.39E-04 | 0.0389 | 0.0065 | 0.1861 | *PES1* |
| 22 | chr22 | 31002992 | 31003227 | 8.52E-04 | 0.0389 | 0.0022 | 0.1240 | *ARFGAP1* |
| 24 | chr3 | 5019495 | 5019877 | 8.93E-04 | 0.0389 | 0.0036 | 0.1331 | *BHLHE40* |
| 23 | chr20 | 61915242 | 61915663 | 8.97E-04 | 0.0389 | 0.0025 | 0.1266 | *H2AJ* |
| 26 | chr15 | 63569614 | 63569719 | 9.55E-04 | 0.0398 | 0.0023 | 0.1249 | *APH1B* |
| 27 | chr16 | 29757319 | 29757360 | 1.19E-03 | 0.0473 | 0.0027 | 0.1266 | *C16orf54* |
| 28 | chr8 | 96280910 | 96281195 | 1.22E-03 | 0.0473 | 0.0080 | 0.2062 | *CFAP418-AS1* |
| 29 | chr4 | 186733332 | 186733590 | 1.36E-03 | 0.0497 | 0.0084 | 0.2126 | *SORBS2* |
| 30 | chr2 | 220299585 | 220299659 | 1.38E-03 | 0.0497 | 0.0029 | 0.1266 | *SPEG* |
| 31 | chr5 | 134734905 | 134735068 | 1.53E-03 | 0.0536 | 0.0031 | 0.1311 | *MACROH2A1* |
| 32 | chr19 | 38747080 | 38747285 | 1.66E-03 | 0.0550 | 0.0034 | 0.1331 | *PPP1R14A* |
| 34 | chr22 | 39096492 | 39096513 | 1.72E-03 | 0.0550 | 0.0035 | 0.1331 | *RFWD3* |
| 33 | chr16 | 74700621 | 74700808 | 1.73E-03 | 0.0550 | 0.0050 | 0.1586 | *JOSD1* |
| 35 | chr1 | 26680591 | 26680641 | 1.85E-03 | 0.0565 | 0.0098 | 0.2414 | *CRYBG2* |
| 36 | chr2 | 233924790 | 233925001 | 1.88E-03 | 0.0565 | 0.0037 | 0.1341 | *INPP5D* |
| 37 | chr19 | 11529948 | 11530138 | 1.97E-03 | 0.0577 | 0.0039 | 0.1341 | *RGL3* |
| 38 | chr11 | 3078611 | 3078852 | 2.05E-03 | 0.0579 | 0.0040 | 0.1341 | *CARS1* |
| 39 | chr17 | 7388064 | 7388450 | 2.11E-03 | 0.0579 | 0.0041 | 0.1341 | *POLR2A* |
| 40 | chr22 | 44577592 | 44577920 | 2.14E-03 | 0.0579 | 0.0056 | 0.1689 | *PARVG* |
| 42 | chr14 | 75745021 | 75745397 | 2.46E-03 | 0.0650 | 0.0061 | 0.1780 | *RAP1GAP2* |
| 41 | chr17 | 2699690 | 2699860 | 2.68E-03 | 0.0692 | 0.0053 | 0.1653 | *FOS* |
| 43 | chr14 | 55493336 | 55493335 | 3.18E-03 | 0.0778 | 0.2094 | 0.7424 | *WDHD1* |
| 44 | chr12 | 6664873 | 6665335 | 3.23E-03 | 0.0778 | 0.0068 | 0.1898 | *IFFO1* |
| 47 | chr7 | 117067137 | 117067416 | 3.26E-03 | 0.0778 | 0.0370 | 0.4611 | *GPR132* |
| 46 | chr3 | 181428378 | 181428563 | 3.31E-03 | 0.0778 | 0.0075 | 0.1977 | *SOX2* |
| 45 | chr14 | 105532013 | 105532030 | 3.38E-03 | 0.0778 | 0.0073 | 0.1977 | *ASZ1* |
| **c) T0-T1 (Response status)** | | | | | | | | |
|  | CHR | Start | End | p-value | FDR (q) | p-value area | Adjusted p-value area | Main genes |
| **1** | **chr8** | **1900192** | **1901041** | **0.00E+00** | **0.0000** | **0.0002** | **0.0416** | ***KBTBD11*** |
| **2** | **chr19** | **38747080** | **38747378** | **0.00E+00** | **0.0000** | **0.0002** | **0.0416** | ***PPP1R14A*** |
| **3** | **chr20** | **61915242** | **61916072** | **0.00E+00** | **0.0000** | **0.0003** | **0.0416** | ***ARFGAP1*** |
| **4** | **chr6** | **25042370** | **25042678** | **4.06E-06** | **0.0012** | **0.0003** | **0.0416** | ***RIPOR2*** |
| **5** | **chr14** | **106321752** | **106322429** | **8.12E-06** | **0.0016** | **0.0003** | **0.0416** | ***FAM30A*** |
| **6** | **chr17** | **48049953** | **48050381** | **8.12E-06** | **0.0016** | **0.0003** | **0.0416** | ***DLX4*** |
| **7** | **chr16** | **28995844** | **28996270** | **1.22E-05** | **0.0020** | **0.0003** | **0.0416** | ***LAT*** |
| **8** | **chr19** | **49993866** | **49994336** | **2.03E-05** | **0.0030** | **0.0003** | **0.0416** | ***SNORD34*** |
| **9** | **chr11** | **67121100** | **67121358** | **2.84E-05** | **0.0037** | **0.0003** | **0.0417** | ***POLD4*** |
| 10 | chr11 | 2019860 | 2020129 | 7.31E-05 | 0.0086 | 0.0005 | 0.0561 | *H19* |
| 11 | chr1 | 230415344 | 230416101 | 1.42E-04 | 0.0139 | 0.0008 | 0.0671 | *GALNT2* |
| 12 | chr6 | 30128239 | 30128542 | 1.42E-04 | 0.0139 | 0.0008 | 0.0671 | *TRIM10* |
| 14 | chr2 | 128458241 | 128458401 | 1.62E-04 | 0.0146 | 0.0008 | 0.0671 | *SFT2D3* |
| 13 | chr5 | 134734905 | 134735104 | 1.79E-04 | 0.0149 | 0.0009 | 0.0671 | *MACROH2A1* |
| 15 | chr17 | 9929529 | 9930101 | 1.95E-04 | 0.0149 | 0.0010 | 0.0671 | *GAS7* |
| 16 | chr22 | 24823390 | 24823554 | 2.07E-04 | 0.0149 | 0.0009 | 0.0671 | *ADORA2A* |
| 17 | chr2 | 233924790 | 233925030 | 2.23E-04 | 0.0149 | 0.0010 | 0.0671 | *INPP5D* |
| 19 | chr11 | 1296470 | 1297087 | 2.32E-04 | 0.0149 | 0.0012 | 0.0695 | *TOLLIP* |
| 18 | chr5 | 139944340 | 139944509 | 2.48E-04 | 0.0149 | 0.0012 | 0.0695 | *SLC35A4* |
| 20 | chr17 | 75471106 | 75471401 | 2.68E-04 | 0.0149 | 0.0010 | 0.0671 | *SEPTIN9* |
| 22 | chr16 | 3174220 | 3174435 | 2.68E-04 | 0.0149 | 0.0016 | 0.0751 | *ZNF205-AS1* |
| 21 | chr3 | 5019218 | 5019877 | 3.13E-04 | 0.0166 | 0.0013 | 0.0707 | *BHLHE40* |
| 23 | chr16 | 29757319 | 29757360 | 3.29E-04 | 0.0167 | 0.0014 | 0.0707 | *C16orf54* |
| 24 | chr11 | 111250287 | 111250431 | 3.66E-04 | 0.0178 | 0.0012 | 0.0695 | *POU2AF1* |
| 25 | chr7 | 124570048 | 124570359 | 4.63E-04 | 0.0208 | 0.0017 | 0.0751 | *POT1* |
| 26 | chr4 | 183061831 | 183062004 | 4.63E-04 | 0.0208 | 0.0017 | 0.0751 | *TENM3* |
| 27 | chr1 | 26644823 | 26645487 | 6.17E-04 | 0.0268 | 0.0020 | 0.0854 | *UBXN11* |
| 28 | chr20 | 57414885 | 57415377 | 7.68E-04 | 0.0321 | 0.0022 | 0.0939 | *GNAS* |
| 29 | chr22 | 39096492 | 39096513 | 8.85E-04 | 0.0357 | 0.0025 | 0.1018 | *JOSD1* |
| 30 | chr8 | 41522722 | 41522935 | 9.46E-04 | 0.0369 | 0.0032 | 0.1193 | *ANK1* |
| 32 | chr12 | 130646023 | 130646256 | 9.87E-04 | 0.0372 | 0.0054 | 0.1572 | *FZD10-AS1* |
| 31 | chr22 | 44577528 | 44577920 | 1.11E-03 | 0.0407 | 0.0030 | 0.1153 | *PARVG* |
| 33 | chr7 | 150498494 | 150498843 | 1.21E-03 | 0.0428 | 0.0036 | 0.1289 | *TMEM176B* |
| 36 | chr22 | 19710092 | 19710280 | 1.43E-03 | 0.0468 | 0.0041 | 0.1345 | *SEPT5-GP1BB* |
| 34 | chr1 | 78511236 | 78511466 | 1.47E-03 | 0.0468 | 0.0037 | 0.1289 | *GIPC2* |
| 35 | chr18 | 3411822 | 3412088 | 1.49E-03 | 0.0468 | 0.0037 | 0.1289 | *TGIF1* |
| 37 | chr1 | 24648852 | 24648984 | 1.49E-03 | 0.0468 | 0.0216 | 0.3215 | *GRHL3* |
| 39 | chr7 | 1014700 | 1014867 | 1.52E-03 | 0.0468 | 0.0042 | 0.1345 | *ADAP1* |
| 38 | chr19 | 2457078 | 2457145 | 1.70E-03 | 0.0511 | 0.0043 | 0.1345 | *LMNB2* |
| 40 | chr1 | 31196413 | 31196527 | 1.90E-03 | 0.0541 | 0.0079 | 0.2055 | *MATN1* |
| 41 | chr17 | 7833655 | 7834014 | 1.90E-03 | 0.0541 | 0.0047 | 0.1442 | *KCNAB3* |
| 44 | chr10 | 97453639 | 97453674 | 2.07E-03 | 0.0567 | 0.0255 | 0.3373 | *TCTN3* |
| 42 | chr6 | 31540412 | 31540750 | 2.08E-03 | 0.0567 | 0.0052 | 0.1549 | *LTA* |
| 43 | chr13 | 110918326 | 110918683 | 2.16E-03 | 0.0573 | 0.0056 | 0.1596 | *COL4A1* |
| 45 | chr7 | 64023703 | 64024074 | 3.22E-03 | 0.0836 | 0.0068 | 0.1880 | *ZNF680* |
| 46 | chr20 | 61427685 | 61427693 | 3.40E-03 | 0.0865 | 0.0070 | 0.1899 | *MRGBP* |
| 47 | chr5 | 141030608 | 141030831 | 3.59E-03 | 0.0894 | 0.0072 | 0.1919 | *FCHSD1* |
| 48 | chr10 | 94050650 | 94050819 | 3.73E-03 | 0.0910 | 0.0338 | 0.3916 | *CPEB3* |
| 49 | chr14 | 55493336 | 55493573 | 4.06E-03 | 0.0969 | 0.0351 | 0.3916 | *WDHD1* |

**Supplementary Figure 1.** Gene set enrichment analyses of genes annotated to significant differentially methylated regions (DMRs) in the longitudinal analysis performed between T0 and T1, including clinical symptoms variations (assessed by delta MADRS) at T1. (A) Top 10 enriched gene sets for gene ontology (GO) molecular functions (MF). (B) Top 10 enriched gene sets for KEGG pathway. (C) Top 10 enriched gene sets for Reactome pathway. (D) Top 10 enriched hallmark gene sets defined by MSigDB (collection H). Dots are coloured according to the adjusted p-value. Dot size represents the number of genes belonging to the gene set/pathway (gene sets with fewer than 2 genes were filtered out).


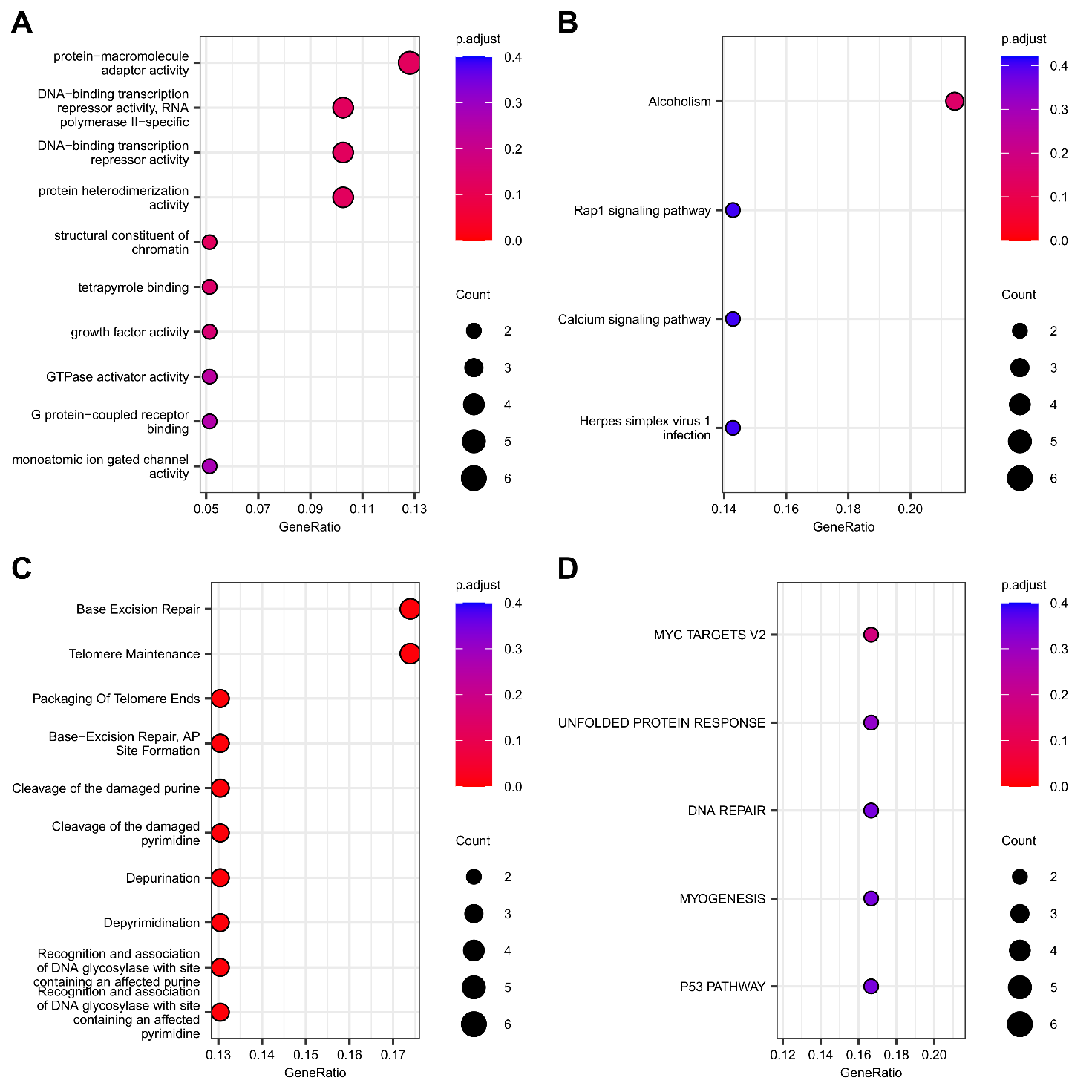


**Supplementary Figure 2.** Gene set enrichment analyses of genes annotated to significant differentially methylated regions (DMRs) in the longitudinal analysis performed between T0 and T1, including clinical symptoms variations (assessed by delta MADRS) at T1. Top 10 enriched Transcription Factor Target gene sets defined by MSigDB for (A) legacy and (B) GTRD subsets (collection C3: regulatory target gene sets, legacy and GTRD subsets).


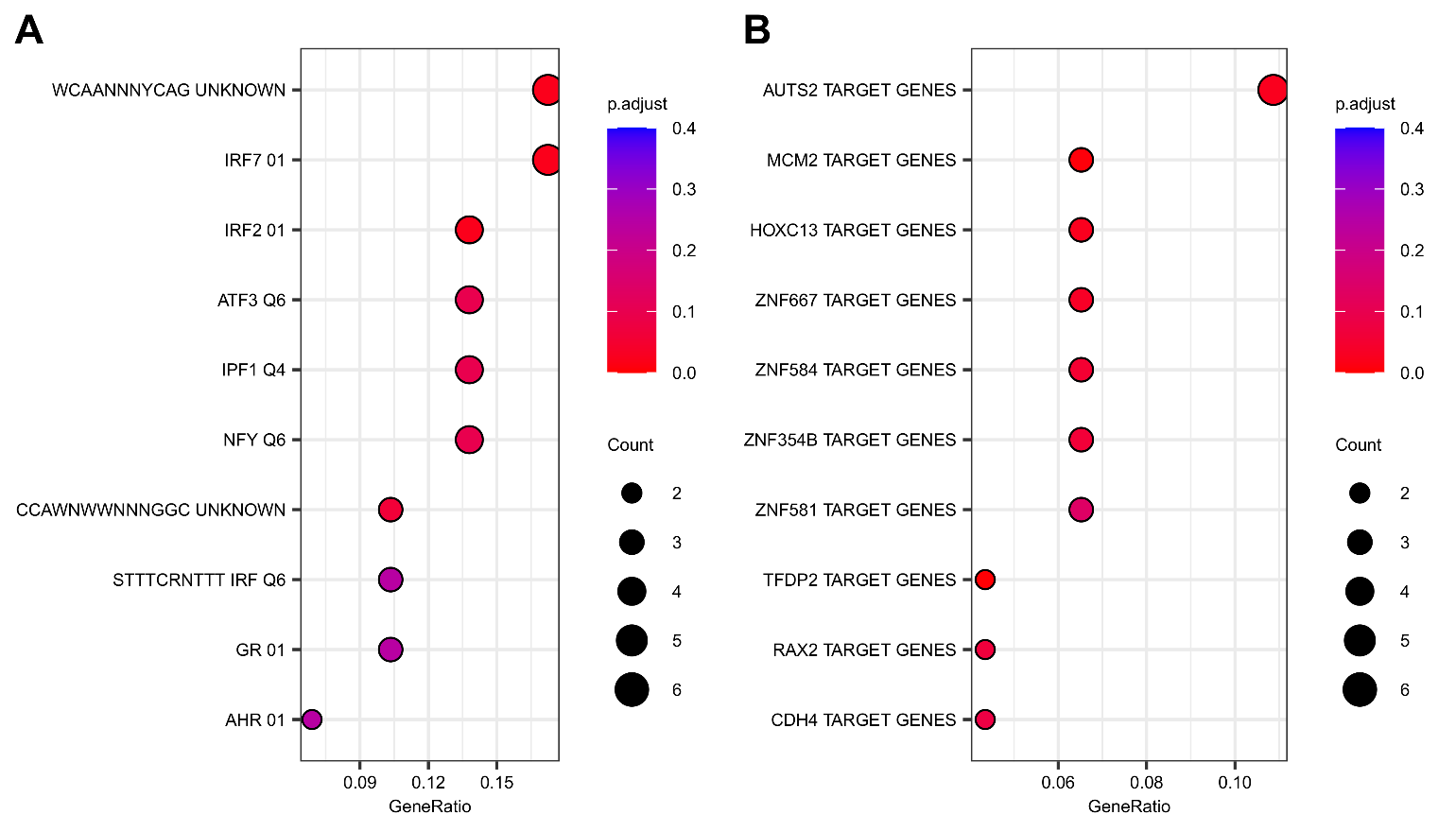


**Supplementary Figure 3.** Gene set enrichment analyses of genes annotated to significant differentially methylated regions (DMRs) in the longitudinal analysis performed between T0 and T1, including response status at T1. Top 10 enriched gene sets for (A) gene ontology (GO) biological processes (BP), (B) GO molecular functions (MF), and (C) GO cellular components (CC). Dots are coloured according to the adjusted p-value. Dot size represents the number of genes belonging to the gene set/pathway (gene sets with fewer than 2 genes were filtered out).

**
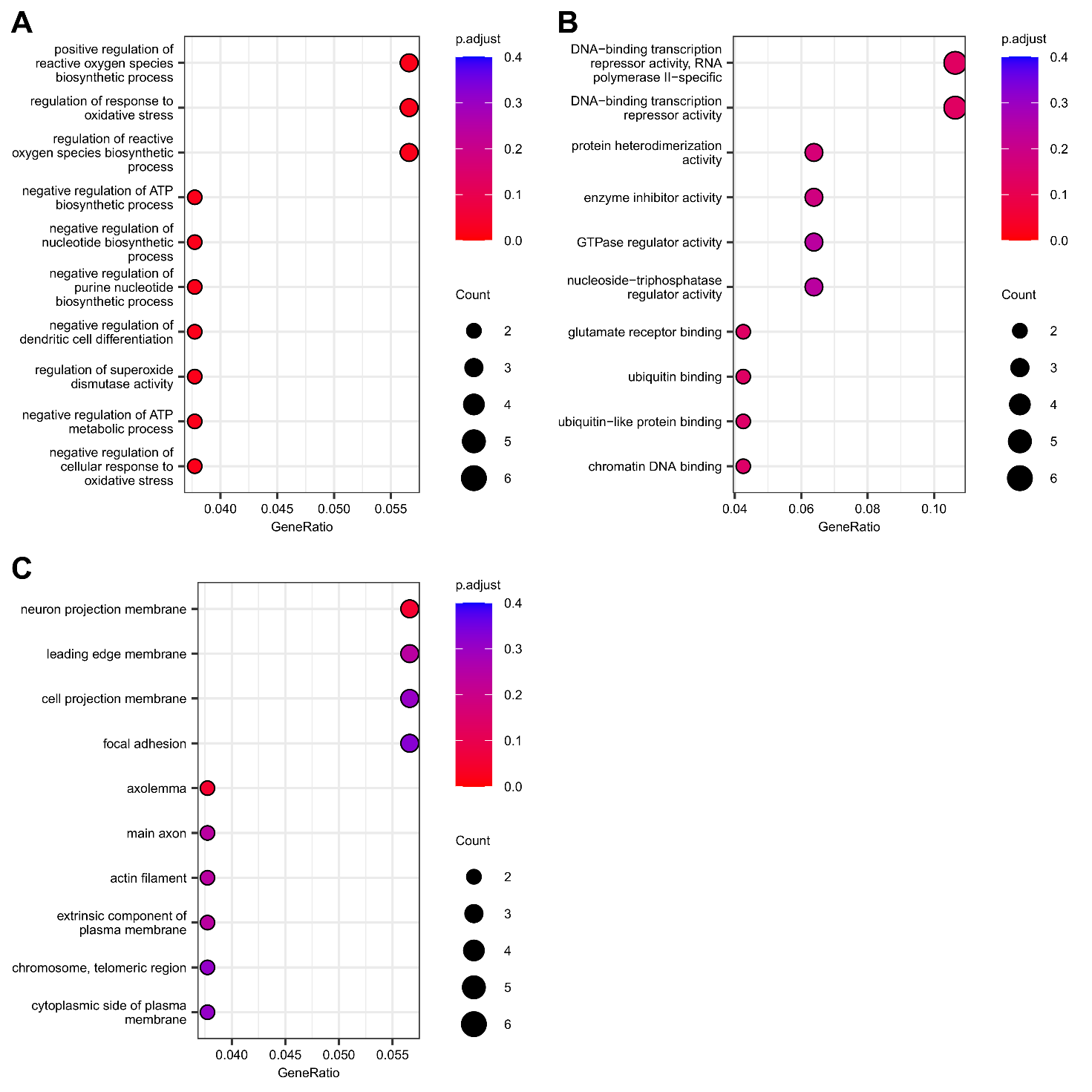
**
